# Functional Determinants and Evolutionary Consequences of Pleiotropy in Complex and Mendelian Traits

**DOI:** 10.1093/molbev/msaf232

**Published:** 2025-09-17

**Authors:** Yury A Barbitoff, Polina M Bogaichuk, Nadezhda S Pavlova, Polina V Malysheva, Alexander V Predeus

**Affiliations:** Institute of Bioinformatics Research and Education, Belgrade 11070, Serbia; JetBrains Research, Belgrade 11070, Serbia; Institute of Bioinformatics Research and Education, Belgrade 11070, Serbia; Institute of Bioinformatics Research and Education, Belgrade 11070, Serbia; Institute of Bioinformatics Research and Education, Belgrade 11070, Serbia; Institute of Bioinformatics Research and Education, Belgrade 11070, Serbia

**Keywords:** genotype-to-phenotype map, pleiotropy, Human Phenotype Ontology, mammalian phenotype ontology

## Abstract

Pleiotropy, a phenomenon of multiple phenotypic effects of the same genetic alteration, is one of the most important features of genotype-to-phenotype networks. Over the last century, biologists have actively debated the prevalence, mechanisms, and consequences of pleiotropy. In this work, we employed data on genotype-to-phenotype associations from the Human Phenotype Ontology and Mouse Genome Database, as well as genome-wide associations from the UK Biobank and FinnGen cohorts to investigate the similarities and dissimilarities in the patterns of pleiotropy between species and different trait types (i.e. Mendelian traits and complex traits). We found that the pleiotropic effects of genes correlate well between species but have a much weaker correlation when comparing different types of traits for the same species. In all cases, however, highly pleiotropic genes possessed a common set of features, such as broad expression across tissues, involvement in many biological processes, or a high number of protein–protein interactions of the respective gene products. Furthermore, we observed a universal tendency of highly pleiotropic genes to be under greater negative selection pressure compared to other groups of genes, including genes essential for cell growth and development. Besides, highly pleiotropic genes also show a significant enrichment of recent positive selection signals. Taken together, our results pinpoint a common mechanism underlying pleiotropic effects in different trait domains and suggest that high degree of pleiotropy plays a role in adaptation, despite imposing additional constraint on genetic variation.

## Introduction

Reconstruction of the complex network of genotype-to-phenotype relationships is a pivotal task of modern genetics. Over the years, the development and application of high-throughput methods have facilitated the construction of large-scale resources that aggregate data on genotype-to-phenotype associations, such as the Mouse Genome Database (MGD) (Blake et al. 2003) or the National Human Genome Research Institute (NHGRI) GWAS Catalog (Buniello et al. 2019). Construction of phenotype ontologies, such as the Human Phenotype Ontology (HPO) (Robinson et al. 2008) and the Mammalian Phenotype (MP) Ontology (Smith et al., 2005), has further enabled accurate integration and comparison of genotype-to-phenotype relationships, both within and between species.

Pleiotropy is a phenomenon in which a single mutation affects multiple independent traits in the phenotype (Wagner and Zhang 2011) and is thus one of the major forces that shape the genotype-to-phenotype networks. Since the advent of genomics, multiple studies have demonstrated the abundance of pleiotropic effects in different species, and different theoretical models of pleiotropy have been proposed. The modular pleiotropy model predicts that genes with similar functions should affect groups (or “modules”) of related traits, while the universal pleiotropy model (also known as the omnigenic hypothesis) states that all genes can be expected to impact all traits (reviewed in Tyler et al. 2016). Recent studies favor the modular pleiotropy model, with two key pieces of supporting evidence: (i) the observed degree of pleiotropy for most mutations is modest, and (ii) the networks of gene–trait connections have a high degree of modularity. For example, both of these features of genotype-to-phenotype networks have been demonstrated in a comparative study of yeast, nematodes, and mice (Wang et al. 2010).

Genome-wide association studies (GWAS) provide a rich set of information for studying pleiotropy in the human genome, and many such studies have been performed (e.g. Pickrell et al. 2016; Chesmore et al. 2018). Emergence of large-scale deeply phenotyped cohorts, such as the UK Biobank (UKB) (Bycroft et al. 2018), has opened new prospects for pleiotropy research. Several efforts have been undertaken to study pleiotropy in UKB (Jordan et al. 2019; Watanabe et al. 2019), including a study performed by our group (Shikov et al. 2020). These studies have confirmed that pleiotropic effects are typical for a substantial proportion of genes, although the estimated proportion of pleiotropic loci varied widely between studies.

Estimation of the degree of pleiotropy from GWAS data is complicated by various factors, including (i) linkage disequilibrium (LD) between multiple genetic variants at a locus, leading to spurious pleiotropy (Chebib and Guillaume 2021); (ii) genetic and environmental correlations between traits that inflate the estimated level of pleiotropy; and (iii) the burden of multiple testing of association between millions of variants and thousands of traits (reviewed in Hackinger and Zeggini 2017). In line with these considerations, the estimated proportion of loci with pleiotropic effects in the UKB data was heavily dependent on the approach used to define independent phenotypes. For example, more than half of the human genome was reported to bear signals of pleiotropy by Watanabe et al. (2019) when using trait domain as a measure of trait independence; however, our analysis based on hierarchical clustering of traits by their phenotypic correlation yielded a much more conservative estimate of 5.4% of the genome (Shikov et al. 2020).

A particularly intriguing question that arises when analyzing pleiotropic effects of genetic variation is the evolutionary consequences of pleiotropy (reviewed in Zhang 2023). In 2000, Orr (2000) found that pleiotropy should naturally decrease the rate of adaptation within Fisher's geometric model framework. This model, however, assumes constant total effects of genetic variants and contradicts empirical observations (e.g. Wagner et al. 2008). Furthermore, Wang et al. (2010) have shown that pleiotropy could, to the contrary, increase the rate of adaptation if the effect size of a mutation is set to be dependent on the degree of pleiotropy. Further support for this model comes from studies that report an enrichment of pleiotropic loci in humans with signals of recent positive selection (e.g. Sakaue et al. 2021). The adaptive effect, however, is expected to be restricted to loci with a moderate degree of pleiotropy, as evidenced by studies in plants (Frachon et al. 2017).

In our earlier work, we have observed an unexpected enrichment of common variants at pleiotropic loci, an observation that challenges the concept of the evolutionary cost of pleiotropy and could possibly indicate an adaptive role of pleiotropic genetic variants. A later study by Novo et al. (2021) suggested that pleiotropic loci are subject to strong background selection; however, the significance of the trend varied for different source data used. Furthermore, the *B* statistic used by Novo et al. can deviate from expectation due to different selection types and may also be confounded by recombination rate. Taken together, results of previous studies do not allow a definitive conclusion regarding the evolutionary consequences of pleiotropy in the mammalian genome.

To carefully address these issues, as well as to provide a deeper insight into the mechanistic basis of pleiotropy in mammals, we have evaluated the pleiotropic effects of human and mouse genes using both Mendelian trait data (provided by the HPO and the MGD) and biobank-scale phenome-wide association studies for hundreds of complex traits (from the UKB and FinnGen projects; Kurki et al. 2023) to analyze functional properties of pleiotropic genes in various species and trait domains.

## Results

### Evaluating the Concordance of Pleiotropic Effects Between Species and Trait Domains

To compare the patterns of pleiotropy between species and trait domains, we first set off to construct a dataset based on publicly available information on genotype-to-phenotype relationships in humans and mice. To this end, we collected and merged data from four main sources: the HPO, the MGD, the pan-UKB, and FinnGen studies. The former two sources contain human (HPO) and murine (MGD) gene annotations with HPO or MP ontologies, respectively, while the latter two datasets represent a compendium of genome-wide association results for 480 quantitative traits and 2,493 complex diseases and disease-related endpoints, respectively (see Methods and Note S1 for details on data preprocessing). In total, the dataset contained 151,636 gene-to-trait relationships, with 52,849, 67,958, 12,065, and 18,764 coming from each of the aforementioned data sources. A total of 4,999, 12,834, 6,469, and 5,650 genes were associated with at least one trait in HPO, MGD, pan-UKB, and FinnGen data, respectively. Of note, only 592 genes had at least one gene–trait association across all four datasets, and trait associations from both trait domains (from either pan-UKB or FinnGen and either HPO or MGD) were available for 4,296 genes. Thus, more than half of the gene–trait connections were unique to each trait domain (55.4% for complex traits data and 68.3% for Mendelian trait data).

**Fig. 1.**
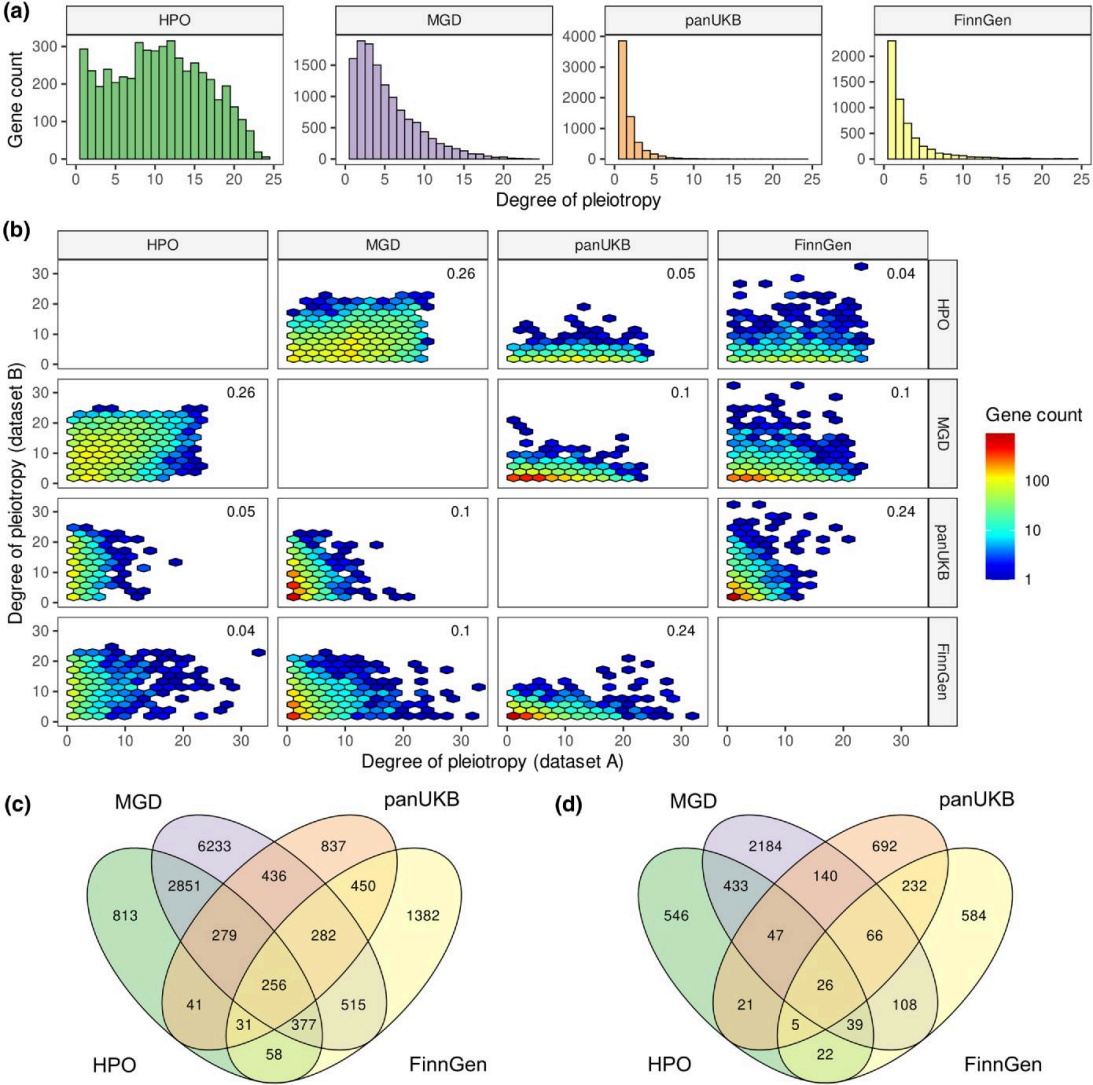
Gene-level degrees of pleiotropy correlate between species and trait domains. a) Distribution of the degree of pleiotropy of human and mouse genes according to different sources of data (from left to right—Human Phenotype Ontology, Mouse Genome Database, pan-UK Biobank, and FinnGen GWAS data). b) Hexagonal scatter plots showing the correspondence between the degree of pleiotropy of genes between different data sources. c, d) Venn diagrams showing the overlaps between sets of all pleiotropic genes c) or highly pleiotropic genes d) between the three datasets (see text for details on cutoff selection).

Before delving into the comparative analysis of the obtained data, we first examined the distribution of the degree of pleiotropy observed in each dataset. While all distributions had a characteristic left skew (Fig. 1a), the overall shape of the distribution varied. The distribution was much more asymmetric in the case of the genome-wide association data (Fig. 1a, two right subplots), with a median of 1 and 2 clusters of correlated complex traits (see Methods for details) associated with a gene. On the other hand, in both HPO- and MGD-based data, the median number of phenotype terms associated with each gene was much greater (11 and 4, respectively), corresponding to a lesser skew of the distribution (Fig. 1a, two left subplots). Only 287 out of 4,999 human genes (5.9%) were associated with exactly one upper-level HPO term, i.e. were nonpleiotropic (for comparison, 59.7% and 40.7% of genes were nonpleiotropic in the two complex trait datasets). In MGD, nonpleiotropic genes comprised a slightly greater fraction of genes (1,605 out of 12,834, 12.5%). In line with the differences in the distribution shape, the genotype-to-phenotype networks differed in the estimated degree of modularity (0.118 and 0.205 vs. 0.516 and 0.550 for HPO and MGD vs. pan-UKB and FinnGen data, respectively), with complex trait-based networks having by far the greatest level of modularity. In all cases, the observed modularity of the network was significantly higher compared to the expected value obtained using random permutation of edges (Extended Data Figure S1).

Having characterized the overall structure of the data, we next went on to compare the patterns of pleiotropy between the datasets. To this end, we used orthologous gene mapping between human and mouse to create a single merged dataset. Comparison of the degree of pleiotropy for single-copy orthologous gene pairs in mouse and human revealed a modest significant correlation (Spearman's *P* = 0.26, Fig. 1b). At the same time, the degree of pleiotropy estimated using complex trait data and Mendelian trait data showed much lower correlation. For instance, there was no significant correlation between the gene pleiotropy in HPO and pan-UKB or FinnGen (Fig. 1b). Remarkably, the degree of pleiotropy estimated for murine genes using MGD data significantly correlated with both GWAS-based estimates for human orthologs (Spearman's correlation = 0.10, *P* < 0.001 in both cases). The greater significance of the estimates for MGD data compared to HPO may be explained by the higher number of genes with nonzero degree of pleiotropy in mice.

Given the relatively low correlation between the degree of pleiotropy estimates, we next tested if a different result could be obtained when testing the overlap between sets of pleiotropic and nonpleiotropic genes without taking into account the numeric estimate of the degree of pleiotropy. To this end, we divided each dataset into four categories: (i) nonpleiotropic genes with no known phenotype (denoted as “NP”); (ii) nonpleiotropic genes associated with exactly one upper-level ontology term or one complex trait cluster (denoted “ST”); (iii) highly pleiotropic (“HP”) genes (identified as top quartile of genes by degree of pleiotropy; the trait/term count cutoffs are 15 [HPO], 9 [MGD], 2 [pan-UKB], and 3 [FinnGen]); and (iv) modestly pleiotropic (“MP”) genes (this group includes all of the remaining pleiotropic genes). The proportions of genes in different groups can be seen on Extended Data Figure S2 and are consistent with the shape of the respective distributions of the degree of pleiotropy (i.e. a greater fraction of genes in MP/HP groups, as well as a lower proportion of ST/NP genes, is observed for GPO and MGD).

We next compared the sets of pleiotropic genes between the dataset. When moderately pleiotropic and highly pleiotropic genes were combined, 2,275 genes were pleiotropic in both trait domains (hypergeometric test *P*-value << 0.011), and a total of 256 genes overlapped between all datasets (Fig. 1c). When only highly pleiotropic genes were considered, 26 genes emerged as overlapping between all data types (Fig. 1d). Importantly, as many as 2,851 genes were pleiotropic according to both HPO and MGD data but were not pleiotropic in GWAS, and a total of 9,897 genes were uniquely pleiotropic according to either HPO or MGD data. This amounts to 81.3% of all genes that were pleiotropic in either human or mouse based on Mendelian trait data. At the same time, only 54.0% (2,669) of genes pleiotropic in pan-UKB or FinnGen data were not pleiotropic in the Mendelian trait domain, with the vast majority of these genes (2,501) having no phenotype associations in either HPO or MGD. Of note, the percentages of shared highly pleiotropic genes was even lower than those observed for all pleiotropic genes (only 13.0% of genes highly pleiotropic in HPO or MGD were pleiotropic in GWAS).

One factor that may substantially affect the observed overlap between sets of pleiotropic genes is the ascertainment bias resulting from different methods of phenotyping used (such an effect is expected to be most pronounced when comparing HPO and MGD datasets, taking into account that phenotyping in controlled experimental conditions could be more thorough and unbiased compared to phenotyping based on self-reporting of patients with Mendelian disease). We hypothesized that, if the ascertainment bias is pronounced, a greater proportion of known essential genes should have a detailed phenotypic description in humans compared to mice (i.e. mild phenotypes should be underrepresented in HPO data). To test this hypothesis, we utilized genes from the full spectrum of intolerance to loss of function (FUSIL) (Cacheiro et al. 2020). In FUSIL, genes are divided into five categories—cellular lethal (CL), developmental lethal (DL), subviable (SV), viable with (VP) or without (VN) phenotype, according to the results of CRISPR screens in human cell lines and deep phenotyping of mice by the International Mouse Phenotype Consortium (IMPC). As expected, a much higher fraction of essential genes (from CL, DL, and SV categories) had associated Mendelian phenotypes; however, this enrichment was significant for both HPO and MGD. Thus, 51.4% of DL genes were associated with at least one term in HPO data (compared to only 17.7% among all human genes) (Extended Data Figure S2). For MGD, a staggering 94.8% of DL genes had phenotype annotations (compared to the baseline value of 44.3%). While these figures emphasize a much richer phenotypic description of murine genes, they do not indicate a substantial bias toward functionally important genes in HPO.

Taken together, these numbers suggest that, despite low correlation between the degrees of pleiotropy between complex and Mendelian traits, there is a significant overlap between the sets of pleiotropic genes between trait domains. It is also important to note that human orthologs of as many as 1,233 genes with pleiotropic effects in MGD, but not HPO data, were pleiotropic according to GWAS data. These findings may suggest that many of the genes that demonstrate detectable effects on the phenotype in mice have a subtle phenotypic manifestation in humans that is detectable only by genome-wide association analysis.

### Pleiotropic Genes Are Characterized by Common Functional Properties

Having split the dataset into different groups of genes depending on their estimated degree of pleiotropy, we next sought to identify shared features of pleiotropic genes between species and trait domains. To this end, we have annotated our dataset with various gene-level properties, such as the summary statistics of gene expression profile (human gene data from GTEx were used), numbers of protein–protein interactions (according to the STRING database), and measures of haploinsufficiency and triplosensitivity (Collins et al. 2022).

**Fig. 2.**
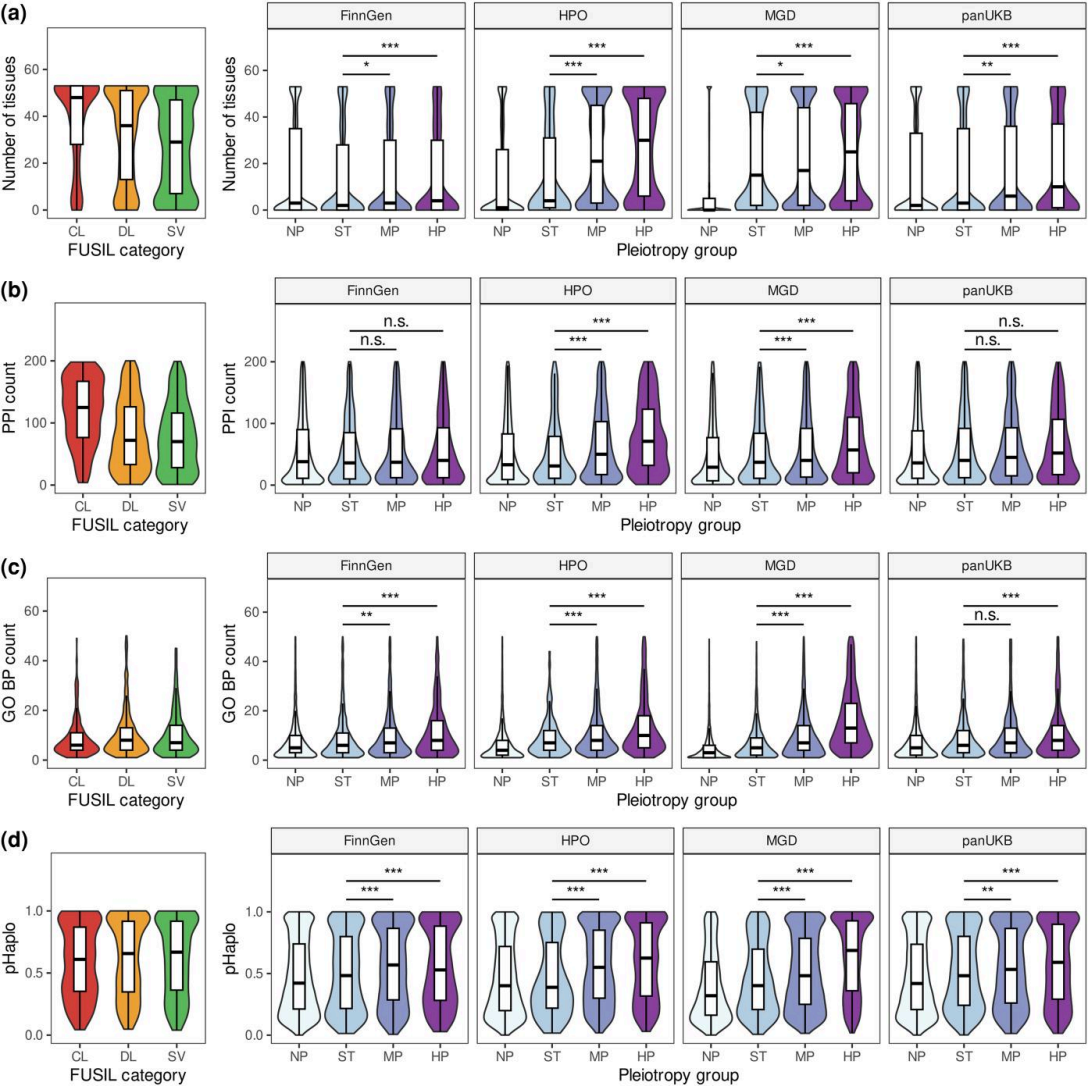
Pleiotropic effects correlate with functional significance of genes in both trait domains. Shown are violin and box plots for a) number of tissues expressing a gene at the level of at least five TPM according to the Genotype Tissue Expression (GTEx) v8 data; b) number of protein–protein interactions according to the STRING database; c) number of biological process (BP) terms from gene ontology (GO); and d) pHaplo scores from Collins et al. 2022. The following abbreviations are used for pleiotropy groups: NP, genes with no associated phenotypes; ST, genes linked to a single trait or term; MP, moderately pleiotropic genes; HP, highly pleiotropic genes. Values for three categories of essential genes (CL, cellular lethal; DL, developmental lethal; SV, subviable) from the FUSIL study (Cacheiro et al. 2020) are shown on each panel. *P < 0.05, **P < 0.01, ***P < 0.001, n.s., no significant differences in Wilcoxon–Mann–Whitney test with Benjamini–Hochberg FDR adjustment.

Analysis of the expression pattern of genes in different pleiotropy groups showed that more pleiotropic genes tend to have broader expression profiles (as evidenced by the number of tissues with expression at the level of at least five transcripts per million [TPM]; Wilcoxon–Mann–Whitney test *P*-value < 0.001 when comparing nonpleiotropic and highly pleiotropic genes in all datasets; Fig. 2a) and greater mean/median expression across tissues (Wilcoxon–Mann–Whitney test *P*-value < 0.001, Extended Data Figure S3). Importantly, the number of tissues with detectable expression was much greater for highly pleiotropic genes in the Mendelian trait data (30 tissues for HPO and 27 for MGD), compared to those identified from GWAS data (9), and was closer to that of known essential genes (48 and 36 for CL and DL categories in FUSIL, respectively; Fig. 2a). It is also important to note that genes with no phenotypic associations showed the greatest tissue specificity across all data sources, except FinnGen (e.g. a gene with no phenotype was expressed in 1, 0, or 2 tissues for HPO, MGD, and pan-UKB, respectively). These numbers suggest that, despite differences in the depth of phenotyping between the datasets, few functionally important genes lack phenotype information across different types of data.

Besides the breadth of expression across tissues, we also observed a significant increase in the maximum expression level of genes with greater degrees of pleiotropy (*P* < 0.001 in all cases, Extended Data Figure S3). The difference between nonpleiotropic and highly pleiotropic genes was the most pronounced for genes at pleiotropic pan-UKB loci (median of highest expression values—22.2, 23.8, 30.0, and 35.6 TPM for NP, ST, MP, and HP groups, respectively). Similarly to the results of the breadth of expression profile, pleiotropic Mendelian trait genes had higher expression levels compared to genes at pleiotropic GWAS loci (55.3 TPM for HPO data and 54.7 for MGD vs. 36.3 for pan-UKB and 26.6 for FinnGen). Of note, murine genes with no phenotype had by far the lowest level of expression (median of the highest expression value = 3.9 TPM), emphasizing a much greater depth of phenotyping in mice.

In addition to broader expression, we also demonstrated that the genes with greater degree of pleiotropy encode proteins with a larger number of protein–protein interactions. This tendency, however, was more apparent in the Mendelian trait domain (median number of interactions for highly pleiotropic proteins = 127 and 101 compared to 42.5 and 45.0 for nonpleiotropic human and murine genes, respectively, *P* < 0.001) (Fig. 2b). For GWAS-based data, no statistically significant differences in the number of PPIs were detected (however, the difference could be seen with a different approach to GWAS data preprocessing, see Note S1, section 2). Again, the number of interactions for highly pleiotropic GWAS genes was much smaller compared to highly pleiotropic Mendelian trait genes, in line with our observations made during expression pattern analysis. For all datasets but HPO, however, highly pleiotropic genes had lower numbers of PPIs compared to essential genes, which was especially high for the CL category (Fig. 2b).

As a next step, we then tested whether the highly pleiotropic genes have broader sets of functions compared to nonpleiotropic ones. To this end, we have annotated each gene with the number of corresponding gene ontology (GO) terms and compared these numbers between gene groups. Indeed, highly pleiotropic genes had higher numbers of associated terms in all three GO branches (biological process [BP], cellular component [CC], and molecular function [MF]) (Fig. 2c, Extended Data Figure S4). Thus, a median highly pleiotropic Mendelian trait gene was involved in 11 and 16 BPs (for human and mouse, respectively, according to HPO and MGD data), compared to only 7 and 5 for nonpleiotropic ones. In the complex trait domain, the difference was also significant (9 vs. 6 for FinnGen, 8 vs. 6 for pan-UKB; *P* << 0.001). In good concordance with the results obtained for other gene-level features, genes with no phenotype information universally had the lowest number of associated GO terms across all data sources (median number from 3 to 5 terms). More curiously, highly pleiotropic genes from all datasets were annotated with a larger number of BP and MF terms compared to essential genes (including CL, DL, and SV genes) (Fig. 2c).

Given the above observations, we also tested whether the highly pleiotropic genes have higher degree of dosage sensitivity. To perform such a test, we employed the pHaplo and pTriplo metrics calculated in a recent study of copy number variation in the human genome (Collins et al. 2022). Indeed, we observed that significantly more pleiotropic genes have higher pHaplo and pTriplo values, approaching and even surpassing those of known essential genes (Fig. 2d, Extended Data Figure S5). The difference in pHaplo and pTriplo was also weaker for complex trait data (HP vs. ST, pHaplo: 0.59 vs. 0.48 in pan-UKB data, 0.62 vs. 0.39 in HPO; pTriplo: 0.50 vs. 0.43 in pan-UKB; 0.54 vs. 0.40 in HPO).

Besides the analysis of various measurable properties of the genes, we also used gene set enrichment analysis to get insights into the function of the genes in different pleiotropy groups. Due to the inherent uncertainty of causal gene identification from GWAS data, we primarily focused this analysis on genes identified using Mendelian trait data (HPO and MGD). For highly pleiotropic genes in both humans and mice, analysis of canonical pathways (CPs) and hallmark gene sets demonstrated enrichment for pathways related to cell proliferation and DNA repair (e.g. KEGG's collection of pathways in cancer, UV response and TGF**β** signaling hallmarks) (Extended Data Figures S6 and S7). Similar results were obtained when analyzing GO term enrichment, with development, morphogenesis, and cell proliferation among the most enriched BP terms (Extended Data Figure S8). In line with these observations, MF term analysis identified terms related to chromatin and DNA binding, transcription factor binding, and protein complex formation to be the most overrepresented (Extended Data Figure S9). Again, these patterns were shared for highly pleiotropic human and murine genes, and both of these sets were highly enriched for CC terms associated with chromatin (Extended Data Figure S10).

**Fig. 3.**
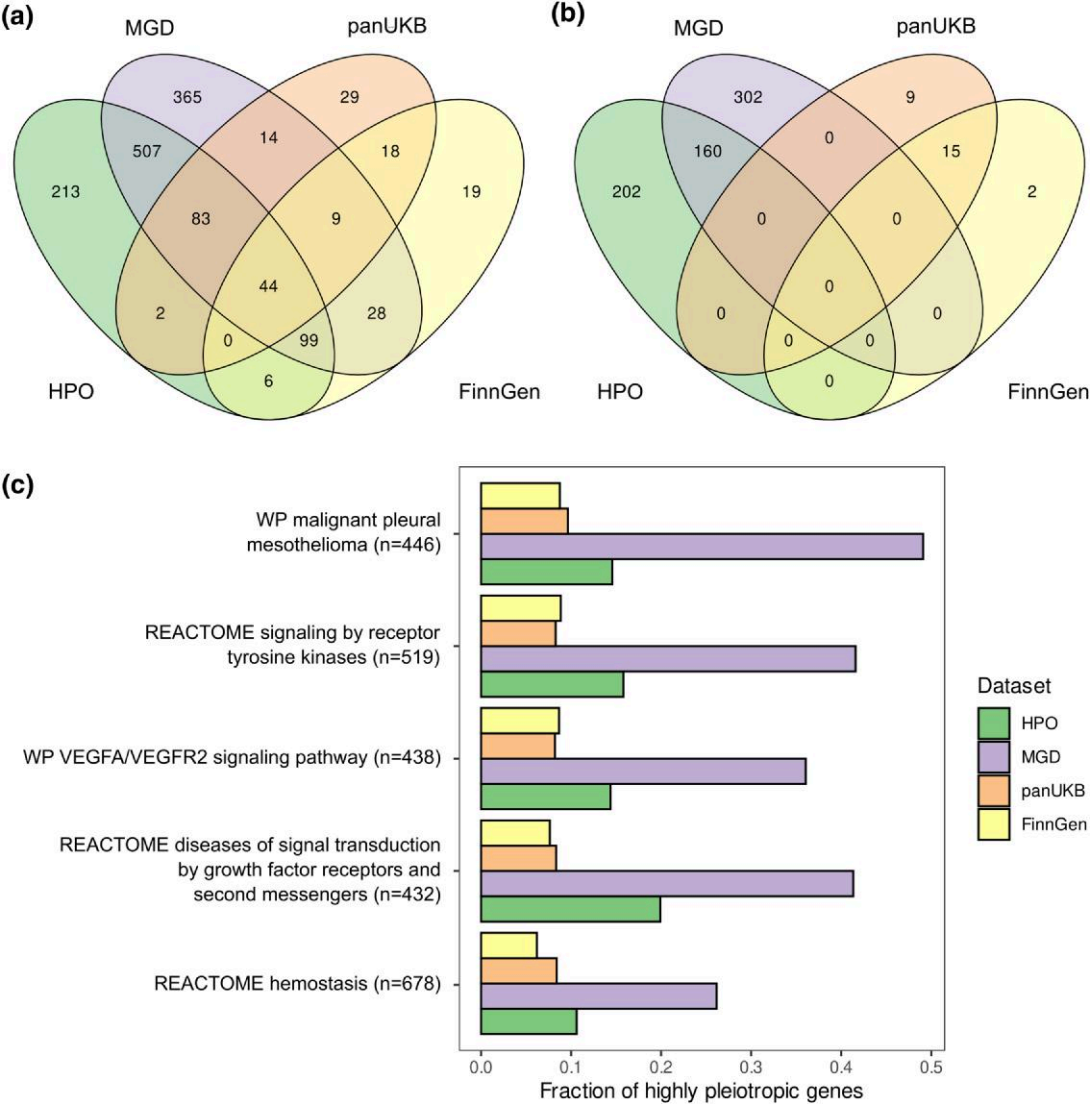
Pleiotropic genes in complex and Mendelian traits are involved in many common processes. a, b) Venn diagrams showing the overlap between enriched GO terms identified using the highly pleiotropic gene sets from indicated data sources. All a) or dataset-specific b) highly pleiotropic genes were used for analysis. c) A bar plot showing the fraction of highly pleiotropic genes for the six large canonical pathways that are significantly enriched with highly pleiotropic genes from all three datasets.

Of note, we also found that highly pleiotropic GWAS genes tend to show enrichment for highly similar gene sets. Thus, 44 enriched MSigDB CPs were shared between highly pleiotropic genes from all four data sources, and only a minor fraction of pathways (66 out of 351, or 18.8%) was uniquely enriched among highly pleiotropic complex trait genes (Fig. 3a). The degree of overlap was even more significant for enriched GO terms, with 733 BP terms shared between all data sources, and only a handful of terms (50 out of 1,915, or 2.6%) were uniquely enriched among pleiotropic GWAS genes. In other GO branches, the proportion of terms specific to complex trait data was also small but slightly higher (17.5% for CC and 13.7% for MF, corresponding to 10 and 22 terms in each case, respectively) (Extended Data Figure S11). These results show that the overlap in the BPs and MFs of highly pleiotropic genes is greater compared to the overlap of the gene sets themselves (Fig. 1d).

We next questioned if the observed overlap in the sets of enriched gene sets is driven by genes that are pleiotropic in both trait domains. To answer this question, we performed gene set enrichment analysis on highly pleiotropic GWAS genes that had no phenotype associations in either HPO or MGD (and vice versa). Importantly, such an analysis identified a large number of terms and pathways enriched among highly pleiotropic genes specific to HPO and MGD data (Fig. 3b). For genes uniquely pleiotropic in GWAS, only a handful of hits were discovered (26 CPs, 5 GO BP terms, and 2 GO MF terms)—for GWAS data (Fig. 3b, Extended Data Figure S11). Of note, no processes or pathways shared between trait domains were identified when using the domain-specific genes for enrichment analysis. This result implies that, while there is a very substantial overlap in the set of BPs controlled by highly pleiotropic genes from Mendelian and complex trait domains, this overlap is likely driven by genes that demonstrate pleiotropic effects in both domains. Domain-specific pleiotropic genes, on the other hand, tend to be involved in a specific set of processes. For the complex trait domain, this set of processes predominantly included various pathways associated with xenobiotic and steroid metabolism.

Having demonstrated the similarity in the sets of processes controlled by pleiotropic genes, we next went on to identify the BPs, CCs, and MFs that are associated with the greatest proportion of highly pleiotropic genes across all datasets. To this end, we focused our analysis on a limited set of GO terms and pathways with large numbers of genes (see Methods for details) and then prioritized these gene sets by the minimum proportion of pleiotropic genes across datasets. Five out of 56 preselected CPs showed significant enrichment of highly pleiotropic genes in all three datasets. In good concordance with results described above, these pathways corresponded to cell proliferation-controlling signaling cascades (e.g. growth factor receptor signaling cascade), genes involved in hemostasis and immune system function (Fig. 3c). In line with these findings, the highest proportion of highly pleiotropic genes was found for GO BP terms related to development, immune cell differentiation and activity, hemostasis, and cell adhesion (Extended Data Figure S12a). Within the CC ontology, pleiotropic genes from both trait domains were involved in transcriptional regulation complexes and chromatin, as well as in cell membrane regions and vesicles (Extended Data Figure S12b). In good concordance with these observations, MF terms linked to transcription factor binding, protein kinase binding or activity, and DNA binding had the highest fraction of highly pleiotropic genes across all datasets (Extended Data Figure S12c). Of note, the proportions of genes with highly pleiotropic effects in mice were universally higher; thus, for the majority of the aforementioned terms, more than half of the corresponding genes had strong pleiotropic effects according to MGD data.

### Shared Patterns of Selection at Pleiotropic Loci Across Datasets

Results of the functional property analysis suggested that genes at pleiotropic loci (at least, within the monogenic trait domain) correspond to broadly expressed genes with a large number of protein–protein interactions. This result predicts that these genes should experience greater pressure of negative natural selection constraining genetic variation within them. At the same time, it is not clear whether these patterns could be expected at pleiotropic GWAS loci. Hence, we next investigated the patterns of natural selection at pleiotropic genes and loci using several widely used measures of both negative (phastCons scores, loss-of-function [LoF] variation, and missense variation statistics from gnomAD) and positive (population-specific integrated haplotype scores [iHS] and the density of recent coalescence events [DRC150]) selection.

**Fig. 4.**
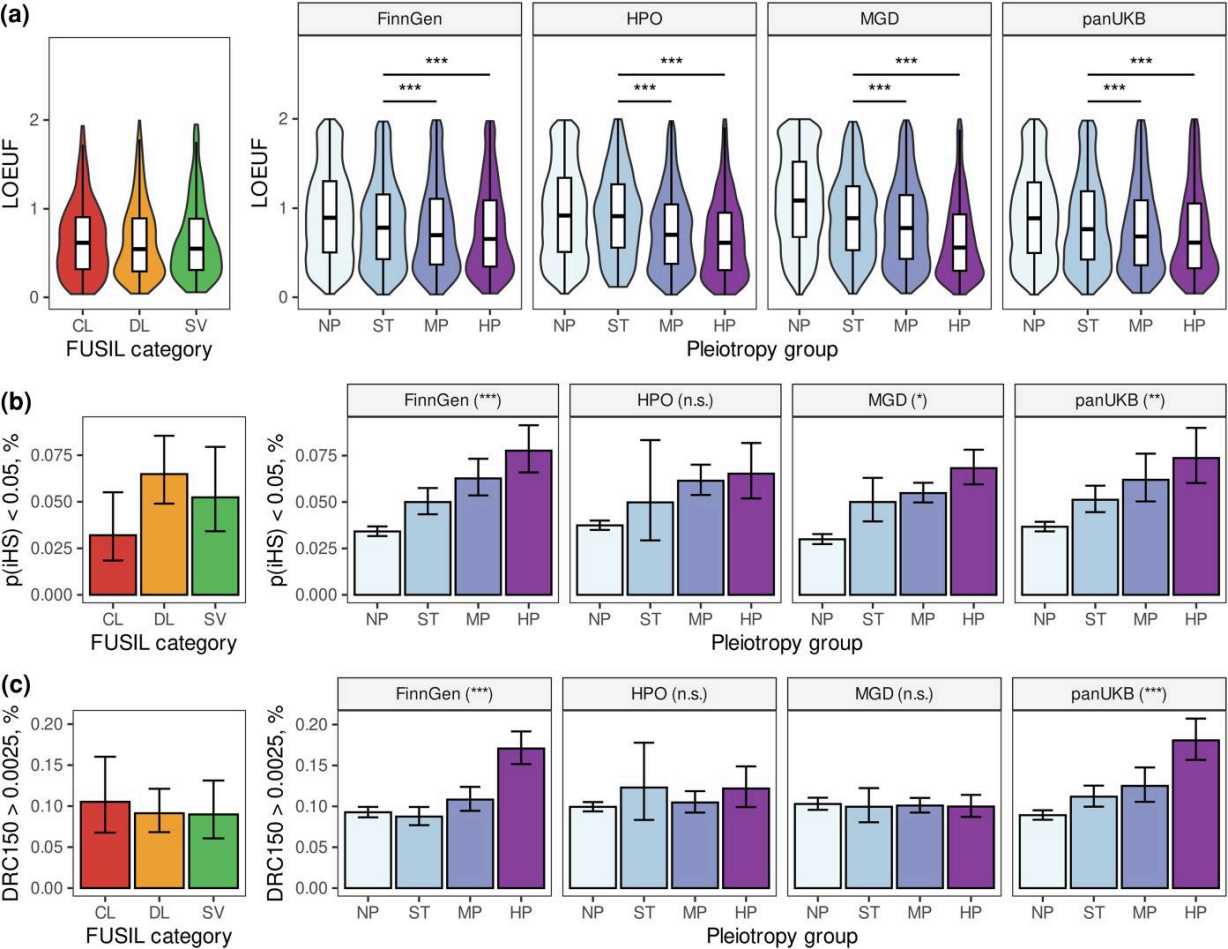
Pleiotropic genes are under greater evolutionary constraint despite bearing signals of recent adaptation. a) Violin-and-box plots of the loss-of-function observed-to-expected upper fraction (LOEUF) values for genes in indicated pleiotropy groups. b, c) Bar plots showing the proportion of genes with statistically significant (DFR-adjusted *P* < 0.01, see Methods) gene-wise maximum iHS value b) or density of recent coalescent events (DRC150) score >0.0025 c) in each pleiotropy group. The following abbreviations are used for pleiotropy groups: NP, genes with no associated phenotypes; ST, genes linked to a single trait or term; MP, moderately pleiotropic genes; HP, highly pleiotropic genes. Values for three categories of essential genes (CL, cellular lethal; DL, developmental lethal; SV, subviable) from the FUSIL study (Cacheiro et al. 2020) are shown on each panel. *P < 0.05, **P < 0.01, ***P < 0.001, n.s., no significant differences in Wilcoxon–Mann–Whitney test (a) or chi-squared test (b, c) with Benjamini–Hochberg FDR adjustment. On (b, c) highly pleiotropic genes were compared to nonpleiotropic ones. Error bars on (b, c) correspond to the 95% confidence interval for the binomial proportion.

When phastCons scores were used as a measure of negative selection, we found no significant difference in the level of evolutionary conservation between pleiotropy groups (Extended Data Figures S13 and S14a,d). However, significant differences between gene groups were detected when using gnomAD-derived gene-level constraint measures (LoF observed-to-expected upper fraction [LOEUF] and probability of LoF intolerance [pLI]). Unlike phastCons scores, which are constructed using whole-genome alignments of multiple distantly related vertebrate species, LOEUF and pLI values reflect the LoF tolerance of human genes based on genetic variation within populations. With these measures being used, highly pleiotropic genes showed universally greater constraint (Fig. 4b, Extended Data Figure S15a). Thus, the median LOEUF value for ST genes was 0.91, 0.89, 0.77, and 0.77 for HPO, MGD, pan-UKB, and FinnGen data, respectively. For highly pleiotropic genes, the value dropped precipitously to 0.61, 0.56, 0.61, and 0.69, respectively. In concordance with some of the earlier observations, this level of constraint was comparable or even more substantial than that of the essential genes (0.61, 0.54, and 0.55 for CL, DL, and SV categories, respectively, from FUSIL).

A similarly pronounced difference was observed when using other population-based metrics of selective constraint, such as the selection coefficient against heterozygous protein-truncating variants such as s_het_ (proposed by Cassa et al. 2017, estimates taken from Zeng et al. 2024, Extended Data Figure S15a) or the *Z*-score of the observed-to-expected missense variant ratio (Extended Data Figure S15b). When genes were split into categories according to their pLI value, highly pleiotropic genes showed a universal and highly significant enrichment with LoF-intolerant (pLI > 0.9) genes across all data sources (30%, 31%, 27%, and 24% of highly pleiotropic genes were LoF intolerant compared to 14%, 13%, 20%, and 20% of nonpleiotropic ST genes for HPO, MGD, pan-UKB, and FinnGen data, respectively; *P* << 0.001 in chi-squared test in all cases). Of note, the proportions of LoF-intolerant genes were similarly high for all FUSIL categories (Extended Data Figure S15c). Taken together, these results indicate that pleiotropic genes are under greater negative selection pressure in both complex and Mendelian trait domains.

We next went on to test the enrichment of pleiotropic genes and loci with recent positive selection signals. As mentioned earlier, two different measures of recent positive selection, which capture different timescales of selection, were utilized. As recent positive selection is not expected to operate uniformly on all genes in a group or across the entire gene sequence, we focused on comparing the proportions of genes for which the maximum value of proximal single-nucleotide polymorphisms (SNPs) either exceeded a predefined threshold or significantly deviated from the gene length-adjusted expectation under the normal distribution (see Materials and Methods for details of the calculation procedure). As can be seen in Figs 4b, c, we observed a general tendency toward the enrichment of pleiotropic genes with signals of recent positive selection, though the strength of this trend varied between the datasets and metrics. For instance, 6.8% of highly pleiotropic murine gene orthologs had a significant maximum iHS value of neighboring SNPs (see Methods for details of the calculation), compared to 3.0% (for the NP group) or 5.0% (for the ST group) of nonpleiotropic ones (Fig. 4c). Similarly, as many as 7.3% and 7.9% of highly pleiotropic genes in the pan-UKB and FinnGen GWAS data bore a recent positive selection signal according to iHS values, compared to only 5.1% and 5.0% of genes with a single associated trait and 3.7% of genes with no associated phenotypes. A similar tendency was observed with using a hard cutoff of iHS > 4 (Extended Data Figure S16a) and was accompanied by a subtle shift in the median value (Extended Data Figure S16b). When the DRC150 value was used as a measure of positive selection, only highly pleiotropic complex trait genes showed a significant enrichment with large DRC150 values (18.0% and 19.4% compared to 11.1% and 9.3% for nonpleiotropic genes, according to pan-UKB and FinnGen data, Fig. 4c).

Given a general trend of highly pleiotropic genes to be longer, especially in GWAS data (Extended Data Figure S17a), we explicitly tested the impact of gene length on the enrichment of recent positive selection signals at pleiotropic GWAS loci. Two methods were employed for this purpose: (i) evaluating the proportion of loci (irrespective of their gene content) bearing a significant selection signal and (ii) comparing the observed proportion of genes with positive selection signals to random sets of genes with comparable length. Enrichment of recent positive selection at highly pleiotropic genes/loci was confirmed with both of these methods for the DRC150, but not iHS (Extended Data Figure S14, Extended Data Figure S17b–e). Of note, the effect of pleiotropy on DRC150 in both pan-UKB and FinnGen data was by far more pronounced when a maximum value of DRC150 for all variants in a 100,000 bp locus was evaluated (36.4% vs. 10.3% [pan-UKB], Extended Data Figure S14c,f).

Given the above observations, we next questioned if highly pleiotropic loci are enriched with genes that bear a combination of negative and positive selection signatures. To answer this question, we selected a subset of human genes with strong selective constraint against LoF variation (LOEUF < 0.6) and a nearby signal of recent positive selection (gene-level FDR-adjusted *P*-value[|iHS|] < 0.05, see Methods). A total of 406 (1.6%) genes met these conditions. The proportion of such genes among nonpleiotropic ones was similar (1.9% in HPO and MGD, 1.9 and 1.8% in GWAS data). Among the highly pleiotropic genes, however, this proportion was significantly higher for all datasets except HPO (4.4%, 3.4%, and 2.9% for MGD, pan-UKB, and FinnGen, respectively) (Extended Data Figure S18a). Similar results were also observed when using a combination of low LOEUF and high DRC150 values (Extended Data Figure S18b).

Finally, we asked if the observed overlap between signals of negative and positive selection can be explained by the general correlation between gene-level constraint (LOEUF) and iHS/DRC150 that may be driven by background selection. To answer this question, we compared the LOEUF values for genes with and without a neighboring positive selection signal (according to iHS or DRC150). This analysis showed that, while genes with a significant maximum iHS value indeed tend to be under greater selective constraint, an inverse tendency is observed for DRC150 (Extended Data Figure S19). Hence, we conclude that the observed enrichment of overlapping signals of negative and positive selection cannot be explained solely by the effects of background selection.

Taken together, these findings demonstrate a universal propensity of pleiotropic genes to be under increased selective constraint and validate earlier suggestions regarding a possible enrichment of pleiotropic loci with positive selection signals. Moreover, our results reveal a unique enrichment of pleiotropic genes with those bearing combined signals of positive and negative selection.

## Discussion

The phenomenon of pleiotropy has attracted the attention of researchers since the early years of genetics. It is now well established that pleiotropy is abundant in natural systems, and the distribution of the degree of pleiotropy usually has a characteristic L shape (reviewed in Zhang 2023). All four data sources used in our analysis also showed a characteristic left skew of the distribution; however, we observed substantial differences in the median degree of pleiotropy as well as the estimated modularity of the underlying genotype-to-phenotype networks. Overall, gene-level pleiotropy estimated using HPO and MGD data was much higher compared to the genome-wide association data. Earlier studies based on mouse phenotype data also showed a higher median degree of pleiotropy (Wang et al. 2010; Muñoz-Fuentes et al. 2022). It is thus necessary to consider the possible sources of these differences.

The most natural explanation of the results is the different nature of traits and mutations used to construct the data. For HPO, the main source of gene annotation is the phenotype observed in patients with Mendelian disease caused by a pathogenic variant in a given gene, usually being a LoF allele (Köhler et al. 2021). Similarly, mouse phenotype data come from single gene or high-throughput knockout assays. Thus, genotype–phenotype relationships recorded in both HPO and MGD have two characteristic features. First, these relationships come from high-impact genetic variants which are commonly assumed to have higher degrees of pleiotropy compared to regulatory variation (Carroll 2005). Second, the phenotypic description in both HPO and MGD predominantly focuses on phenotypic abnormalities observed in affected individuals. In both of these regards, HPO and MGD substantially differ from complex trait data. For example, genetic variation used to conduct genome-wide association analysis is mostly regulatory or silent, though pleiotropic variants are known to be enriched for missense variants (Watanabe et al. 2019; Shikov et al. 2020). Another distinctive feature of the phenome-wide association data is that these datasets typically include a more diverse set of traits (including continuous ones). However, it is important to note that we did not find any marked differences in the patterns of pleiotropy observed in pan-UKB and FinnGen data, even though different types of traits were used for analysis from these two sources (continuous traits and complex diseases, respectively). Besides, murine quantitative trait loci (QTL) data from MGD also show a distribution of the degree of pleiotropy similar to those observed for different types of human complex traits (see Note S1, section 4).

While the trait and mutation type could explain the observed differences in the degree of pleiotropy, it is hard to rule out the possibility of a technical confounding. For example, upper-level MP ontology terms are not entirely independent, with possible implications for pleiotropy analysis (Muñoz-Fuentes et al. 2022). In our analysis, we chose upper-level MP terms over other ontology term-based metrics as they maximized the number of nonpleiotropic genes and minimized the median degree of pleiotropy (see Note S1, section 1), It is also noteworthy that the genotype-to-phenotype networks constructed using upper-level ontology terms show a significantly higher degree of modularity compared to random networks of similar size (Extended Data Figure S1), suggesting that much of the vertical pleiotropy (pleiotropic effects resulting from cause–effect relationships or shared mechanistic basis of the two traits) has been efficiently removed from the data. However, we believe that the phenotype definition still plays a role in inflating the degree of pleiotropy in certain cases.

For genome-wide association data, on the other hand, the estimate of the gene-level degree of pleiotropy could be impacted by the causal gene selection method based on summary statistics (see Note S1, section 2), as well as method for independent phenotype definition (Note S1, section 3). In our earlier work, we have used phenotypic correlation-based clustering to reduce the impact of vertical pleiotropy, as we have previously shown a positive impact of this procedure on the phenome-wide analysis results (Shikov et al. 2020). While this procedure efficiently reduces the observed degree of pleiotropy, it is still possible that many complex traits with relatively low observed phenotypic correlation could still fall under the same upper-level phenotype ontology term. Hence, we could expect that mapping complex traits to phenotype ontology terms could further reduce the observed statistical pleiotropy in GWAS data.

In our study, we showed that genes bearing highly pleiotropic variation are more broadly expressed (Fig. 2a, Extended Data Figure S3), have more protein–protein interactions (Fig. 2b) and a richer set of MFs (Extended Data Figure S4), participate in more BPs (Fig. 2c), are more dosage sensitive (Fig. 2d, Extended Data Figure S5), and, finally, are under greater selective constraint (Fig. 4a, Extended Data Figure S15). In many of these aspects, highly pleiotropic genes tend to be similar to the ones known to be essential for cellular viability and embryonic development. These results are in line with earlier observations made in model organisms (He and Zhang 2006) and in human complex traits (Shikov et al. 2020). A more intriguing finding, however, is the striking similarity in the properties of highly pleiotropic genes in complex traits and Mendelian traits, which is observed despite the aforementioned discrepancies in the degree of pleiotropy. Besides the consistent effect of pleiotropy on the various gene-level features across datasets, it is also important to emphasize that a highly pleiotropic gene according to GWAS data was more similar to a moderately pleiotropic or nonpleiotropic gene according to HPO or MGD data. These observations are in line with a lower median degree of pleiotropy observed in the genome-wide association data. Nevertheless, we can conclude that pleiotropic effects in both complex and Mendelian trait domains are associated with the same genic features and are hence likely to be guided by similar or overlapping mechanisms.

It is also worth noting that the differences in all gene-level metrics tested were usually much more pronounced in MGD than in HPO data, and certain trends (e.g. the enrichment of highly pleiotropic genes with recent positive selection signals) were significant in MGD, but not HPO. These observations suggest that the phenotype of high-impact genetic variation is much better described in mice than in humans. Indeed, large-scale phenotyping efforts such as the IMPC (Meehan et al. 2017; Cacheiro et al. 2019) have greatly enhanced the depth of mouse phenotype description. In humans, on the other hand, description of the phenotype is performed only by examining the patients with rare disease. Hence, many genes may lack phenotype annotation due to various factors, such as survival of the patient until the time of molecular diagnostics or the ability to clearly demonstrate the causal effect of a genetic variant observed in a patient. However, despite a potentially substantial role of the aforementioned phenotype ascertainment bias, two pieces of indirect evidence suggest that it does not interfere with the analysis of functional and evolutionary gene properties. First, genes with no associated Mendelian phenotypes are much more similar to nonpleiotropic than to pleiotropic genes and are typically showing lower levels of biological relevance and evolutionary constraint compared to single trait-associated genes (Figs 2 and 4). Second, while there is a notable overrepresentation of well-phenotyped genes among the ones known to be essential for development and cell viability (Extended Data Figure S2), this trend is observed for both human and murine genes.

Besides shared properties of pleiotropic genes, our analysis identified a set of processes that are enriched with pleiotropic genes across species and trait domains. This set of pathways includes ones involved in cell proliferation, signaling, immune system function, and extracellular matrix organization (Fig. 3, Extended Data Figure S11). These findings are in good concordance with our earlier analysis of pleiotropic gene functions in complex traits (Shikov et al. 2020). Besides, the present analysis indicates that the previously observed enrichment of highly pleiotropic loci with liver-specific genes is the unique feature of the complex trait domain. Thus, xenobiotic metabolism and related pathways were enriched among GWAS-specific highly pleiotropic genes, but not among highly pleiotropic Mendelian trait genes in either species.

**Fig. 5.**
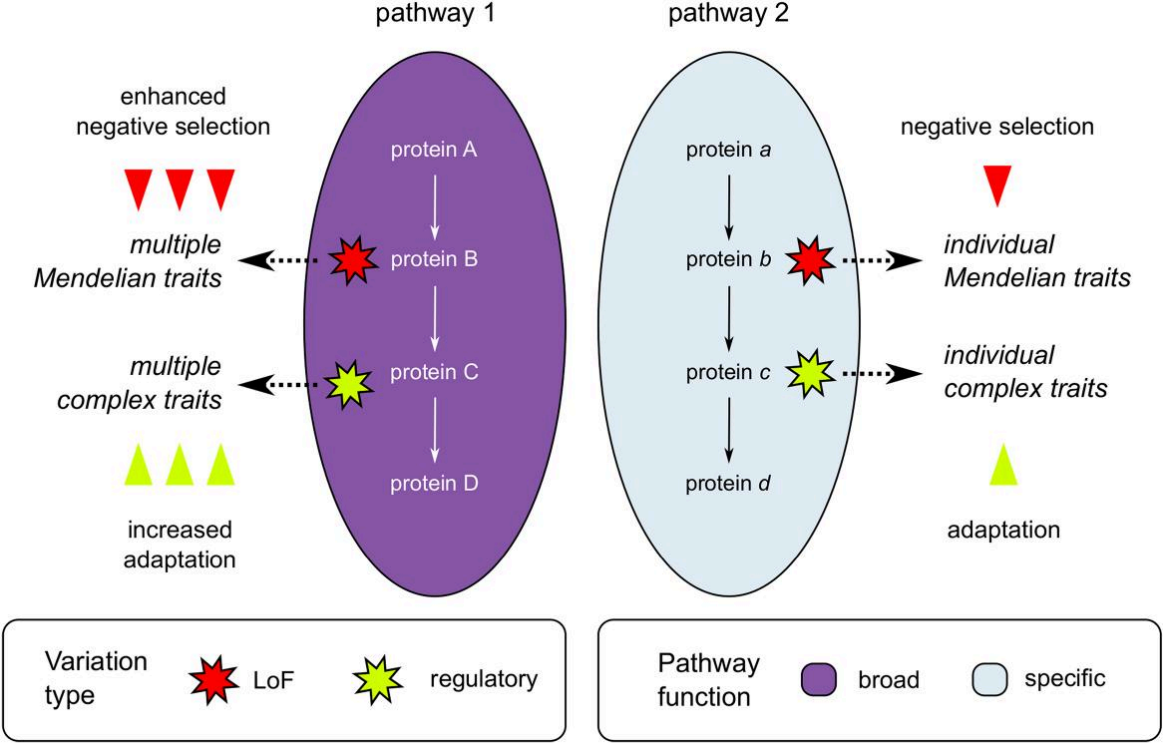
A model for pleiotropic effects in different trait domains. Ellipses represent pathways colored by their functional relevance in an organism (i.e. broad or tissue/cell type specific); arrows connecting proteins within pathways represent the direction of signal transduction or position of genes in metabolic reaction chains. Polygonal stars represent genetic variants that are colored according to their type (LoF or regulatory). See text for further explanation of the model.

Shared functions of highly pleiotropic genes across trait domains (Fig. 3, Extended Data Figure S10) suggest that a highly similar set of pathways is responsible for pleiotropic effects of genetic variation; however, the magnitude of these effects would be dependent on the type of genetic variant (e.g. null [LoF] mutation, change in the amino acid sequence of a protein, or a variant in the regulatory element) (Fig. 5). Thus, high-impact genetic variants in crucial pathway genes would have pronounced pleiotropic effects and manifest in Mendelian phenotypes (rare congenital disorders) and would be subject to enhanced purifying selection. Lower-impact variants in the same genes would also have pleiotropic effects, albeit with a lower magnitude, driving multiple complex traits rather than Mendelian phenotypes. Such variants will be enriched in adaptive alleles, in accordance with both our (Fig. 4) and earlier findings (e.g. Frachon et al. 2017; Sakaue et al 2021). In pathways with a restricted set of functions, on the other hand, all types of mutations would likely lack pleiotropic effects and would hence be both less adaptive and subject to lower negative selective pressure.

In our previous work, we observed an interesting tendency of pleiotropic genetic variants in GWAS to have higher allele frequency compared to their nonpleiotropic counterparts (Shikov et al. 2020). At the time, we have proposed three explanations for this observation: (i) removal of rare variants at pleiotropic loci due to strong purifying selection; (ii) greater power to detect genome-wide associations for common genetic variants; and (iii) spurious pleiotropy of common variants that tag different rare causal variants at the same locus. In addition to the three hypotheses mentioned above, one might also suggest that higher frequency of pleiotropic variants may be driven by an enrichment of loci with recent positive selection signals, an assumption that has been supported by evidence in some recent studies (Frachon et al. 2017; Sakaue et al. 2021). The results included in the present study indicate that pleiotropy correlates both with increased strength of purifying selection and higher frequency of adaptive alleles, favoring both the first and fourth hypotheses. Moreover, our results show that both negative selection strength and the enrichment with positive selection signals are highest for highly pleiotropic genes. This is especially true for genes with pleiotropic effects in mice, which showed both the strongest negative selection against LoF variants and the most pronounced enrichment with adaptive alleles (according to the iHS value). These observations contradict earlier findings and predictions that pleiotropy should increase the rate of adaptation only for moderate degrees of pleiotropy (Wang et al. 2010; Frachon et al. 2017). It is important to note, however, that the enrichment with more recent positive selection (detected using the DRC150 statistic) was observed only in GWAS data, where the cutoff for selection of highly pleiotropic genes was lower. Still, the proportion of genes with high DRC150 value increased when higher degree of pleiotropy was demanded (e.g. 32 out of 168 genes had a positive selection signal when requiring at least eight trait clusters). Hence, we believe that our results emphasize that a high degree of pleiotropy is, at the very least, compatible with active positive selection.

To sum up, pleiotropy is not only a basic feature of genotype-to-phenotype networks, but an inevitable consequence of the organismal complexity. Still, while pleiotropy imposes additional constraint on genetic variation, it appears to provide additional opportunities for adaptation of complex organisms to changing environments.

## Materials and Methods

### Genotype-to-Phenotype Data Collection

To compile a dataset consisting of genotype-to-phenotype relationships in the Mendelian trait domain, we obtained information about orthologous gene pairs between mice and humans from the MGD (the HMD_HumanPhenotype file was used; https://www.informatics.jax.org/downloads/reports/HMD_HumanPhenotype.rpt). For each ortholog, we included associations with phenotype terms from the MP ontology for mice and the HPO for humans. For human genes, gene annotations were downloaded directly from the HPO website (https://hpo.jax.org/data/annotations, accessed on 2024 July 01). For MP data, phenotypic information (excluding conditional mutations) was retrieved from the MGI_GenePheno file (https://www.informatics.jax.org/downloads/reports/MGI_GenePheno.rpt).

As a source of genotype-to-phenotype relationship information in complex trait domains, we utilized publicly available summary statistics of genome-wide association analysis by the pan-UKB study (Karczewski et al. 2025) and FinnGen project release 12 (Kurki et al. 2023). The pan-UKB manifest file with heritability estimates was preprocessed to select traits with *P*(*h*^2^ = 0) *<* 0.01 according to the standard normal distribution (*Z*-score > 2.32). For traits with different encoding and preprocessing methods used in the original pan-UKB study, a single entry with the maximum heritability estimate was selected. Only continuous traits and biomarkers were included into the analysis. Summary statistics files were downloaded for the resulting set of 480 traits (Table S1). As the heritability estimates were not available for FinnGen, summary statistics for all traits were used, excluding quantitative endpoints (BMI, height, and weight).

### Definition of Pleiotropy in Phenotype Ontology Data

To calculate the degree of pleiotropy of a gene, all phenotype ontology terms associated with a particular gene were converted to upper-level terms using the ontobio package (for HPO data, terms were converted to MP to simplify further cross-species comparison, official upper-level term mapping provided by MGI was used: https://github.com/mgijax/mammalian-phenotype-ontology/tree/main/mappings). Due to a strong bias in the number of genes annotated with the MP:0010768 (“mortality/aging”) term, this term was removed from further analysis. Then, the number of remaining upper-level MP terms linked to each gene was used as a measure of its degree of pleiotropy. To identify highly pleiotropic genes, we split all genes into quartiles according to their degree of pleiotropy and then considered all genes in the fourth quartile as highly pleiotropic ones. Besides the aforementioned approach, clustering of all phenotype ontology terms was attempted as a measure of pleiotropy, with results of the analysis being similar to those obtained with upper-level terms (see Note S1, section 1 for details).

### Definition of Pleiotropy in Genome-Wide Association Data

Summary statistics of pan-UKB GWAS were used to identify lead SNPs using the clumping procedure available in PLINK v. 1.9 (Purcell et al. 2007) and the 1000 Genomes phase 3 genotype data (Auton et al. 2015). Following clumping, the closest gene was retrieved for each of the identified lead SNPs using BEDTools (Quinlan and Hall 2010) and the GENCODE v19 human gene annotation file (Frankish et al. 2021 ). An alternative approach based on all genes within a 100,000 bp interval around each lead SNP was also attempted, with similar results (see Note S1, section 2).

A matrix of gene–trait associations was constructed using the inferred causal gene information. Results of hierarchical trait clustering using the phenotypic correlation estimates provided by the authors of the pan-UKB study were used to merge associations by cluster (for each cluster, a gene was considered to be associated with the cluster if it was identified as the closest gene for a lead SNP in GWAS results for at least one trait). The number of associated trait clusters was used as the resulting measure of the degree of pleiotropy, similar to our earlier work (Shikov et al. 2020). Similar to the analysis of phenotype ontology terms, the top 25% of all genes with the highest degree of pleiotropy were considered as highly pleiotropic, and all other genes with a degree of pleiotropy greater than 1 were considered as moderately pleiotropic.

For FinnGen data, the UCSC liftOver toolkit was used to convert genomic coordinates to the hg19 genome assembly. After that, genes closest to lead SNPs were identified using the same procedure as described above for the pan-UKB data, and the gene–trait association matrix was constructed. As an alternative way of causal gene identification, results of fine mapping by SuSIE (Zou et al. 2022) provided by the authors (https://docs.finngen.fi/finngen-data-specifics/green-library-data-aggregate-data/core-analysis-results-files/finemapping-results-format) were employed, and a gene corresponding to a variant with the most severe impact in each credible set was used for further processing. After construction of the gene–trait association matrix, traits were clustered using a distance metric based on reconstituted phenotypic correlation (*d* = 1−*r_p_*). The *r_p_* values were obtained from variant effect sizes and standard errors using the PhenoSpD package for R (Zheng et al. 2018).

### Analysis of Genotype-to-Phenotype Network Modularity

To perform an analysis of modularity of the bipartite gene–trait network, preprocessed gene-to-trait associations from each data source were converted into an incidence matrix. The matrix was then used to construct a bipartite graph using the igraph package (Csárdi and Nepusz 2006), followed by greedy community detection and calculation of network modularity score.

To obtain the distribution of expected modularity scores, each network was randomly shuffled, preserving the total number of edges for each node. This procedure was repeated multiple times, resulting in 100 reshuffled networks for each data source. Modularity was then computed for each such network, and the distribution of the resulting scores was compared with the true modularity of the real network.

### Comparison of Functional Gene Properties

Summary statistics of gene expression profile (median expression across tissues, number of tissues with expression at five or more TPM, maximum expression across tissues) were calculated using the Genotype Tissue Expression (GTEx) v8 data (Lonsdale et al. 2013). The number of protein–protein interactions recorded for a gene was retrieved using the BioGRID database (Oughtred, et al. 2021). Additionally, gene-level probabilities of dosage sensitivity (pHaplo and pTriplo) were taken from a paper by Collins et al. (2022). Finally, the number of GO (Ashburner et al. 2000) terms associated with each gene was calculated using the org.Hs.eg.db and org.Mm.eg.db packages for R.

Besides the analysis of numeric features, a gene set enrichment analysis using the GO term enrichment was performed for highly pleiotropic genes using the clusterProfiler package for R (Yu et al. 2012). In addition to GO terms, hallmark gene sets and CPs from the Molecular Signatures Database (MSigDB) (Liberzon et al. 2011, 2015) were used in gene set enrichment analysis.

For the analysis of gene sets with the most consistent enrichment across datasets, we merged the results of enrichment analysis for the three data sources into a single dataset (per each gene set group: hallmarks, CPs, BP, CC, and MF branches of GO) and then ordered the gene sets by the minimum percentage of highly pleiotropic genes across the data sources. For GO terms, only large (>500 genes) terms were considered. For a largely redundant CP collection, a set of mostly nonoverlapping pathways was constructed as follows: (1) all pathways were ordered by the number of genes; (2) the largest pathway was selected, and all genes corresponding to this pathway were removed; and(3) the procedure was repeated with the remaining genes until at least one pathway with >25 genes remained. This procedure resulted in a set of 56 (out of initial 2,982) mostly nonredundant pathways covering more than 87% (11,681/13,343) of all human genes with known function (Table S2).

To provide a broader context in functional gene property analysis, results of the FUSIL study were employed (Cacheiro et al. 2020). Distributions of the functional metrics were evaluated for three groups of essential genes—CL, DL, and SV. Proportions of pleiotropic genes in different FUSIL categories were also evaluated to assess the strength of phenotype ascertainment bias.

### Signals of Positive and Negative Selection

For the analysis of purifying selection for pleiotropic and nonpleiotropic loci, gene-level constraint measures for human genes were retrieved from the Genome Aggregation Database (gnomAD) v.2.1. (Karczewski et al. 2020). LOEUF, *Z*-score for observed-to-expected missense variant ratio, and the pLI (Lek et al. 2016) were used for gene annotation and comparison. Additionally, we used phastCons (Siepel et al. 2005; 10.1101/gr.3715005) conservation scores computed using 100 vertebrate species obtained from the UCSC Table Browser (https://genome.ucsc.edu/cgi-bin/hgTables, accessed on 2024 July 10). The phastCons scores in bigWig format were converted to bedGraph format using the bigWigToBedGraph tool (https://github.com/ENCODE-DCC/kentUtils, accessed on 2024 July 20) and then averaged over the gene or locus interval.

To assess the overlap with recent positive selection signals, two main metrics were employed that capture different time periods: (i) the integrated haplotype score (iHS) and (ii) density of recent (over the last 150 generations) coalescence events (DRC150). The iHS values for the British (GBR) subpopulation from 1000 Genomes project (Auton et al. 2015) were taken from a recent analysis by Johnson and Voight (2018). DRC150 were taken from the original study by Palamara et al. (2018). For both metrics, gene-wise iHS and DRC150 value were calculated by intersecting the gene interval (for HPO/MP data) or the 100,000 bp interval around the lead SNP with SNP coordinates (annotated with respective iHS and DRC150 values), and the maximum absolute value of each metric was retrieved for a gene or locus in question.

To determine the statistical significance of the gene-wise maximum iHS value, a *P*-value was computed assuming that the iHS value for each of the *n* SNPs in a gene is normally distributed, as follows:


$$p(x|n)=1-\overset{n}{\underset{i=1}{\prod }}\;N(x),$$

where *x* is the observed maximum iHS value and *N*(*x*) is the normal cumulative distribution function.

## Supplementary Material

msaf232_Supplementary_Data

## Data Availability

All data and code pertinent to the analysis presented in this work are available through GitHub: https://github.com/ibre-research/pleiotropy_analysis/.
