# Supplementary material for: Functional Determinants and Evolutionary Consequences of Pleiotropy in Complex and Mendelian Traits
Source: Mol Biol Evol. 2025 Sep 17;42(10):msaf232. doi: 10.1093/molbev/msaf232 (PMC12534788; doi:10.1093/molbev/msaf232)
Supplement: msaf232_Supplementary_Data [file msaf232_supplementary_data.zip › 2025-09-07 - MBE paper Supplementary.pdf]

Functional determinants and evolutionary consequences of pleiotropy in complex and Mendelian traits

Yury A. Barbitoff[1,2], Polina M. Bogaichuk[1], Nadezhda S. Pavlova[1],Polina V. Malysheva[1], Alexander V. Predeus[1]

[1] - Institute of Bioinformatics Research and Education, Belgrade Serbia
[2] - JetBrains Research, Belgrade, Serbia

Correspondence: Yury A. Barbitoff, barbitoff@bioinf.institute

Extended Data

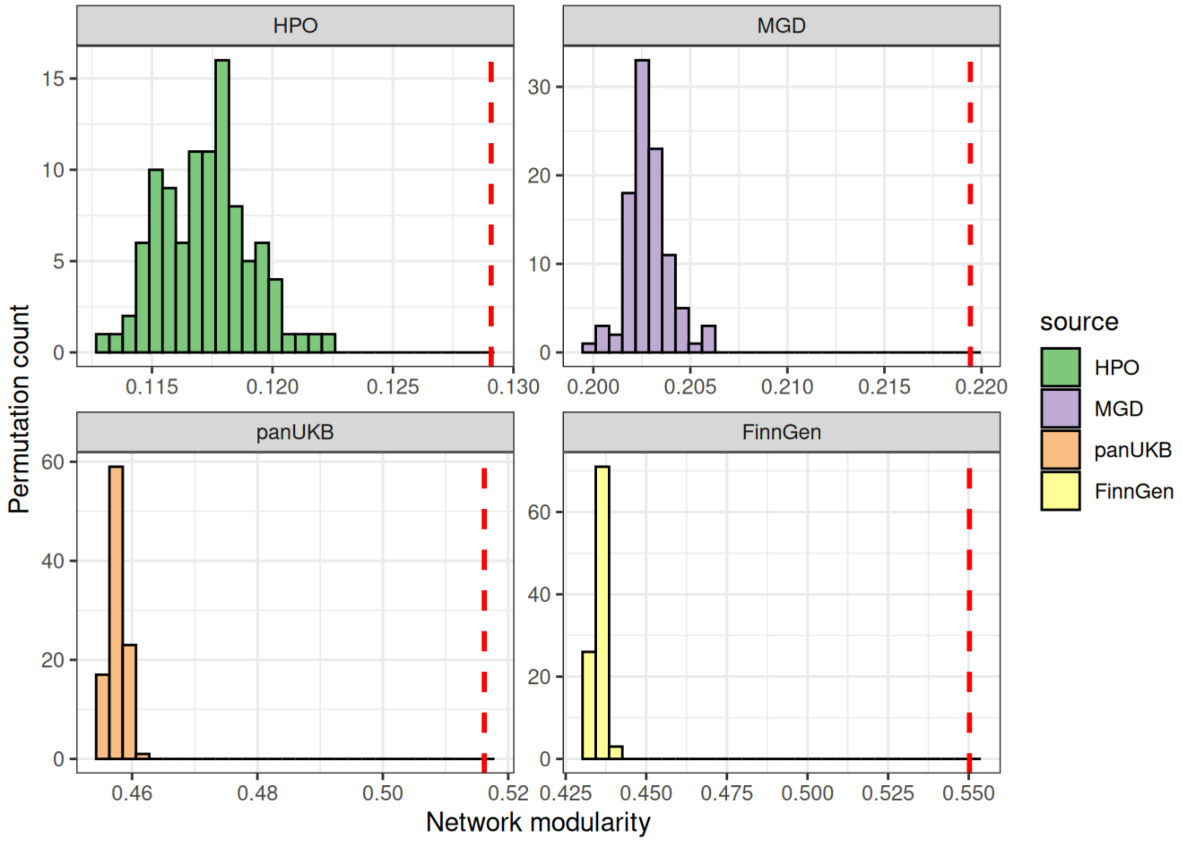

Extended Data Figure 1. Genotype-phenotype networks for all datasets except FinnGen show higher modularity compared to random networks. Shown are histograms of modularity scores (see Methods for details) obtained using randomly permuted genotype-phenotype networks for each source. The dashed read lines correspond to the observed modularity of the real networks.

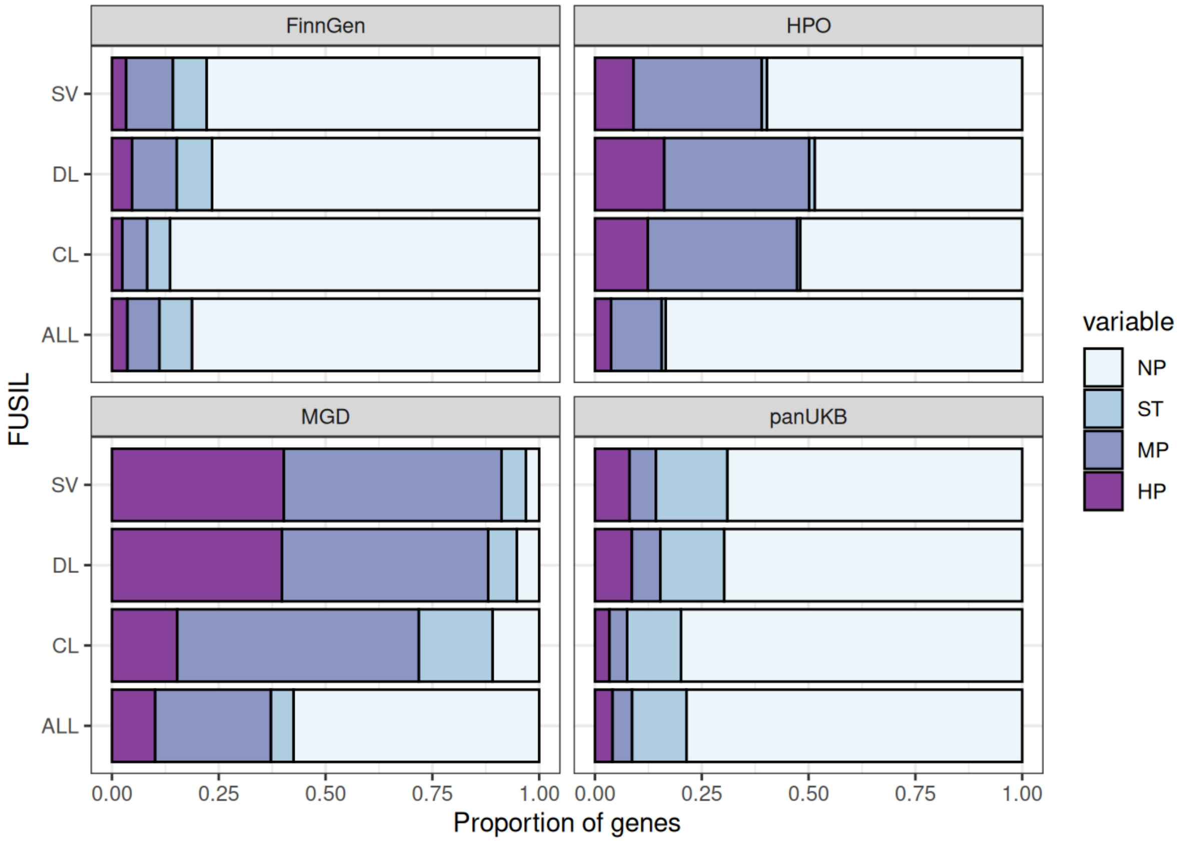

Extended Data Figure 2. Proportion of genes corresponding to different pleiotropy groups and FUSIL gene essentiality categories. The following abbreviations are used for pleiotropy groups: NP - genes with no associated phenotypes, ST - genes linked to a single trait or term, MP - moderately pleiotropic genes, HP - highly pleiotropic genes. FUSIL categories are abbreviated as CL (cellular lethal), DL (developmental lethal), and SV (subviable). ALL - proportions for all genes across the genome.

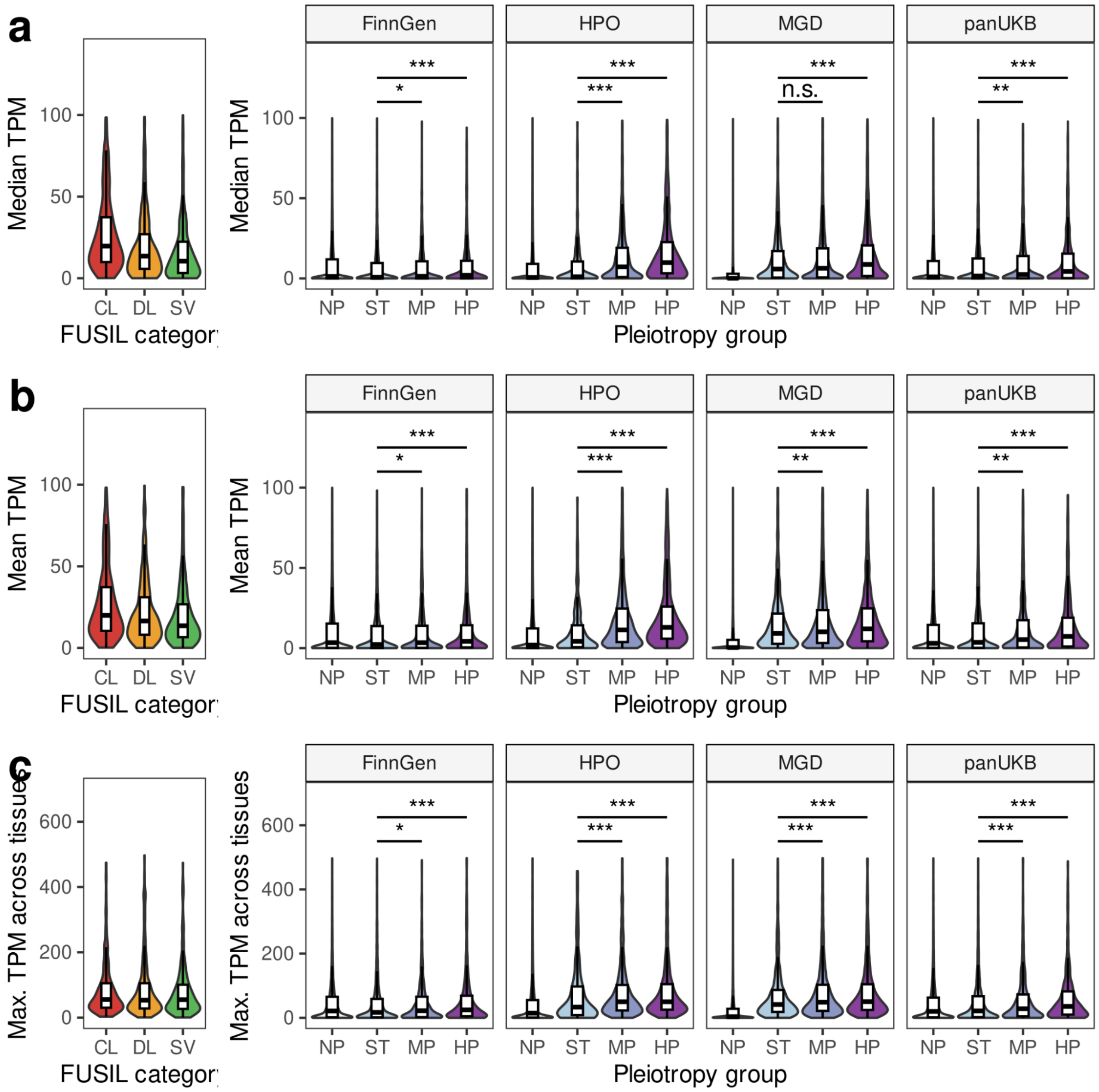

Extended Data Figure 3. Pleiotropic genes have higher expression levels. Shown are violin plots of the median (a), mean (b), and maximum (c) per-tissue expression level of the genes in indiciated pleiotropy groups. All expression values are in transcripts per million (TPM) according to the Genotype Tissue Expression (GTEx) data * - $p < 0.05$, ** - $p < 0.01$, *** - $p < 0.001$ in Wilcoxon-Mann-Whitney rank sum test with Benjamini-Hochberg FDR adjustment. NP - genes with no associated phenotypes, ST - genes linked to a single trait or term, MP - moderately pleiotropic genes, HP - highly pleiotropic genes. FUSIL gene essentiality categories are abbreviated as CL (cellular lethal), DL (developmental lethal), and SV (subviable).

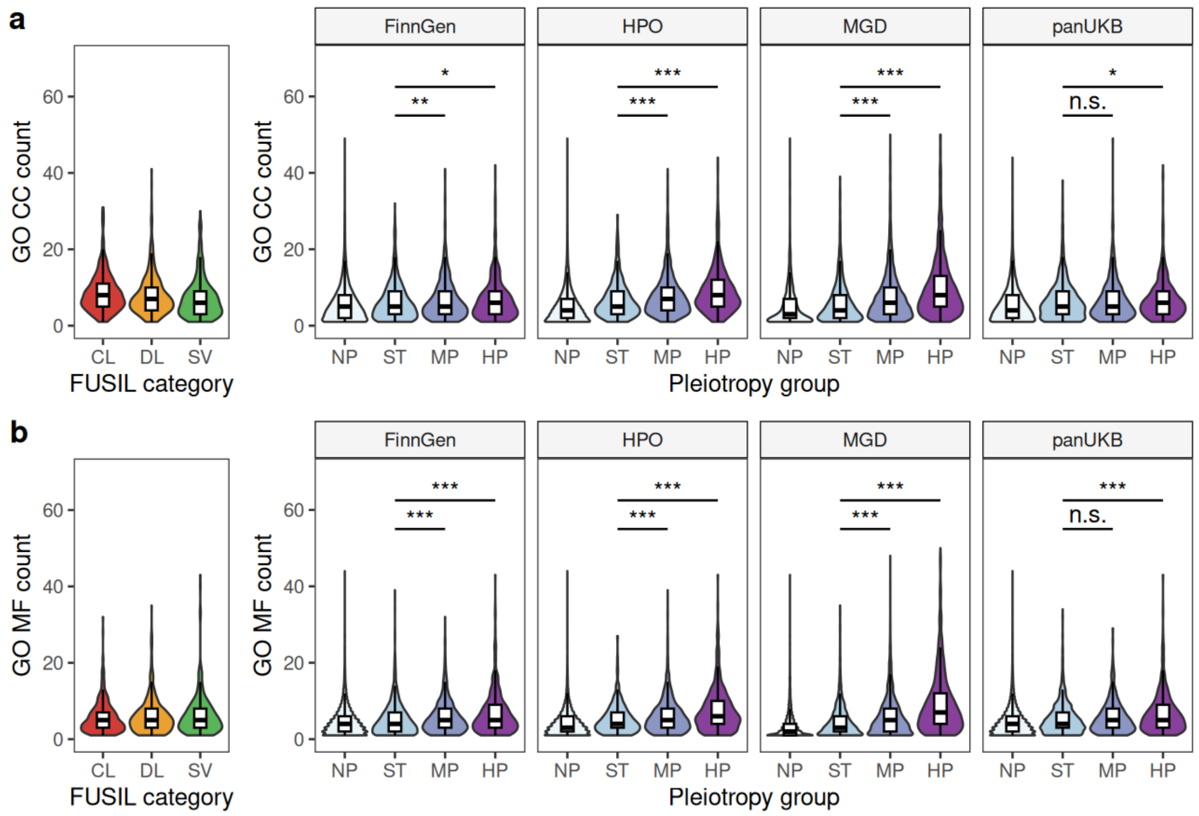

Extended Data Figure 4. Highly pleiotropic genes have more biological functions. Shown are violin plots of the number of cellular component (CC) (a) and molecular function (MF) (b) terms associated with genes in indiciated groups. * - p < 0.05, ** - p < 0.01, *** - p < 0.001 in Wilcoxon-Mann-Whitney rank sum test with Benjamini-Hochberg FDR adjustment. NP - genes with no associated phenotypes, ST - genes linked to a single trait or term, MP - moderately pleiotropic genes, HP - highly pleiotropic genes. FUSIL gene essentiality categories are abbreviated as CL (cellular lethal), DL (developmental lethal), and SV (subviable).

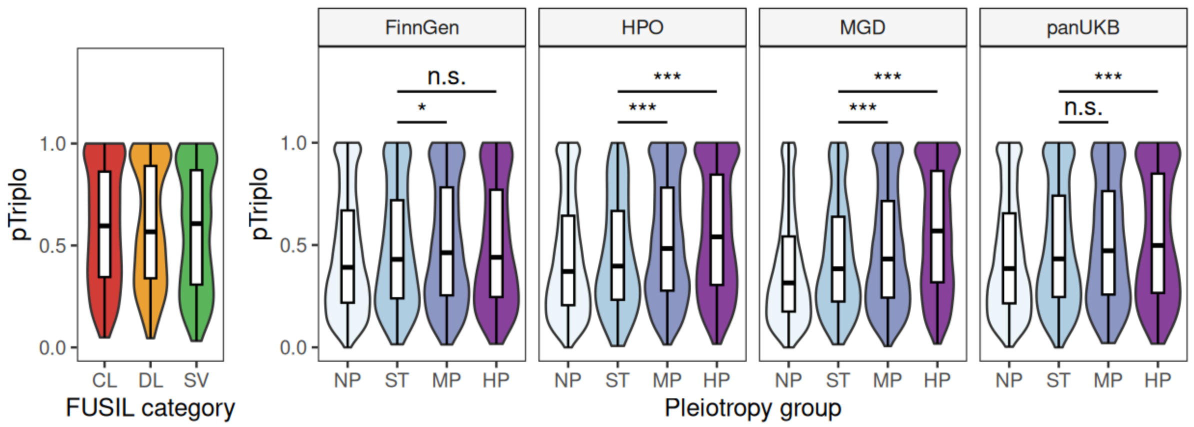

Extended Data Figure 5. Highly pleiotropic genes have higher levels of triplosensitivity. Shown are violin plots and box plots of the probability of triplosensitivity (pTriplo) according to data in Collin *et al*., 2022, ** - $p < 0.01$, *** - $p < 0.001$ in Wilcoxon-Mann-Whitney rank sum test with Benjamini-Hochberg FDR adjustment. NP - genes with no associated phenotypes, ST - genes linked to a single trait or term, MP - moderately pleiotropic genes, HP - highly pleiotropic genes. FUSIL gene essentiality categories are abbreviated as CL (cellular lethal), DL (developmental lethal), and SV (subviable).

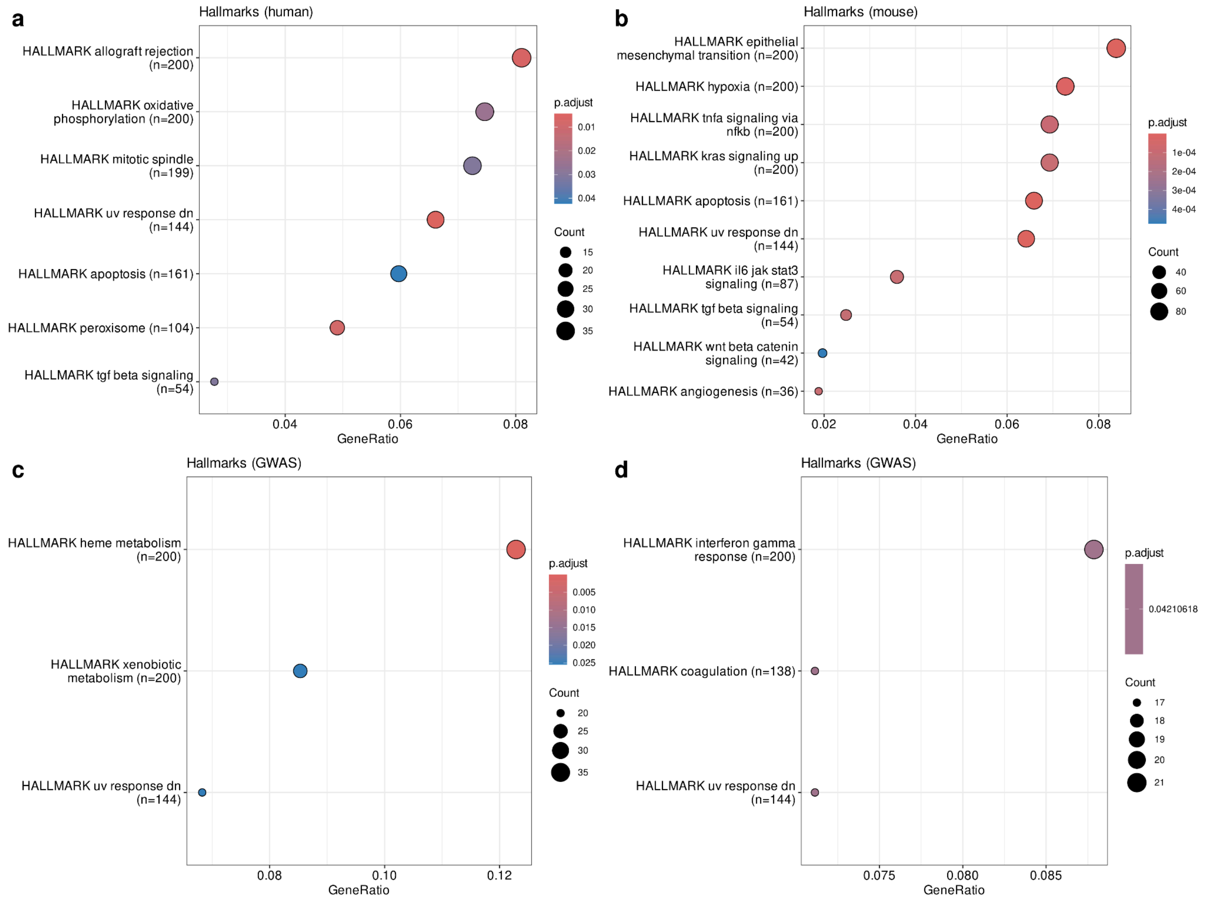

Extended Data Figure 6. Hallmark gene sets associated with high degree of pleiotropy. Shown are dot plots of the gene set enrichment analysis results of highly pleiotropic genes according to HPO (a), MGD (b), and pan-UK Biobank (c) and FinnGen (d) GWAS data. The analysis was performed against hallmark gene sets from MSigDB. Top 10 terms are shown in each case.

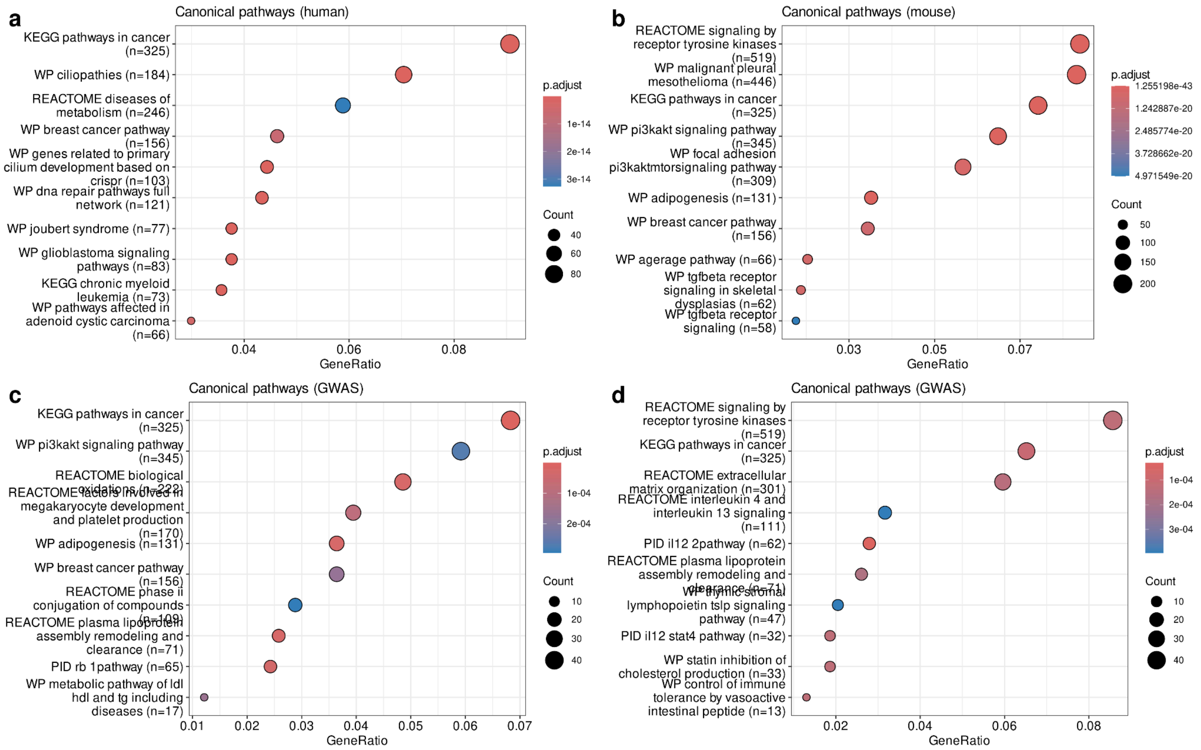

Extended Data Figure 7. Canonical molecular pathways associated with high degree of pleiotropy. Shown are dot plots of the gene set enrichment analysis results of highly pleiotropic genes according to HPO (a), MGD (b), pan-UK Biobank (c), and FinnGen (d) GWAS data. The analysis was performed against canonical pathway (C2:CP) gene sets from MSigDB. Top 10 terms are shown in each case.

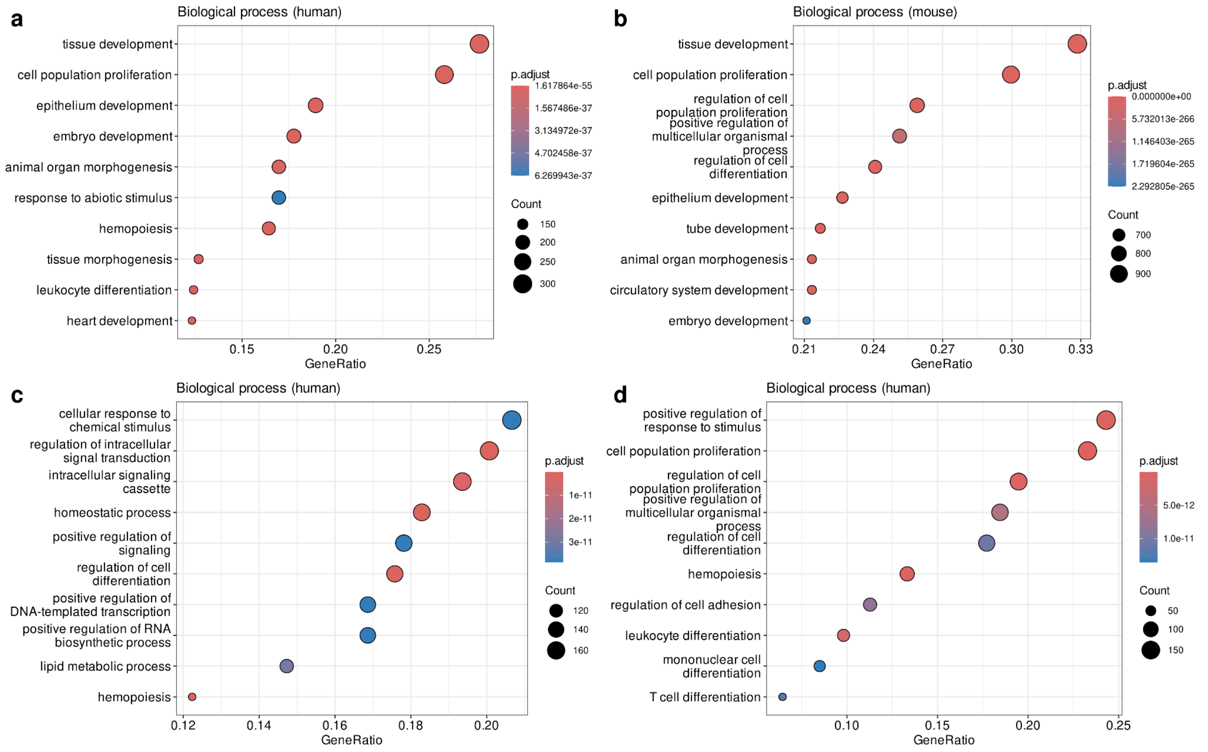

Extended Data Figure 8. Biological processes associated with high degree of pleiotropy. Shown are dot plots of the gene set enrichment analysis results of highly pleiotropic genes according to HPO (a), MGD (b), pan-UK Biobank (c) and FinnGen (d) GWAS data. The analysis was performed against GO biological process terms. Top 10 terms are shown in each case.

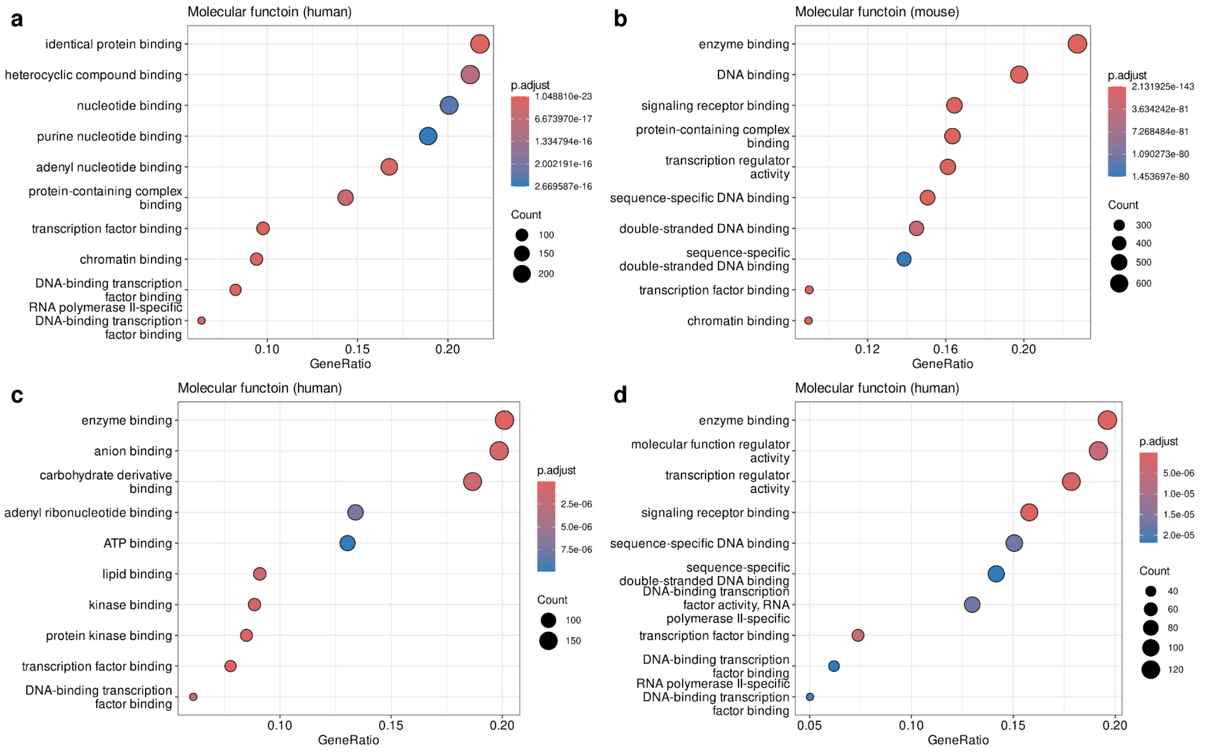

Extended Data Figure 9. Molecular functions associated with high degree of pleiotropy. Shown are dot plots of the gene set enrichment analysis results of highly pleiotropic genes according to HPO (a), MGD (b), pan-UK Biobank (c) and FinnGen (d) GWAS data. The analysis was performed against GO molecular function terms. Top 10 terms are shown in each case.

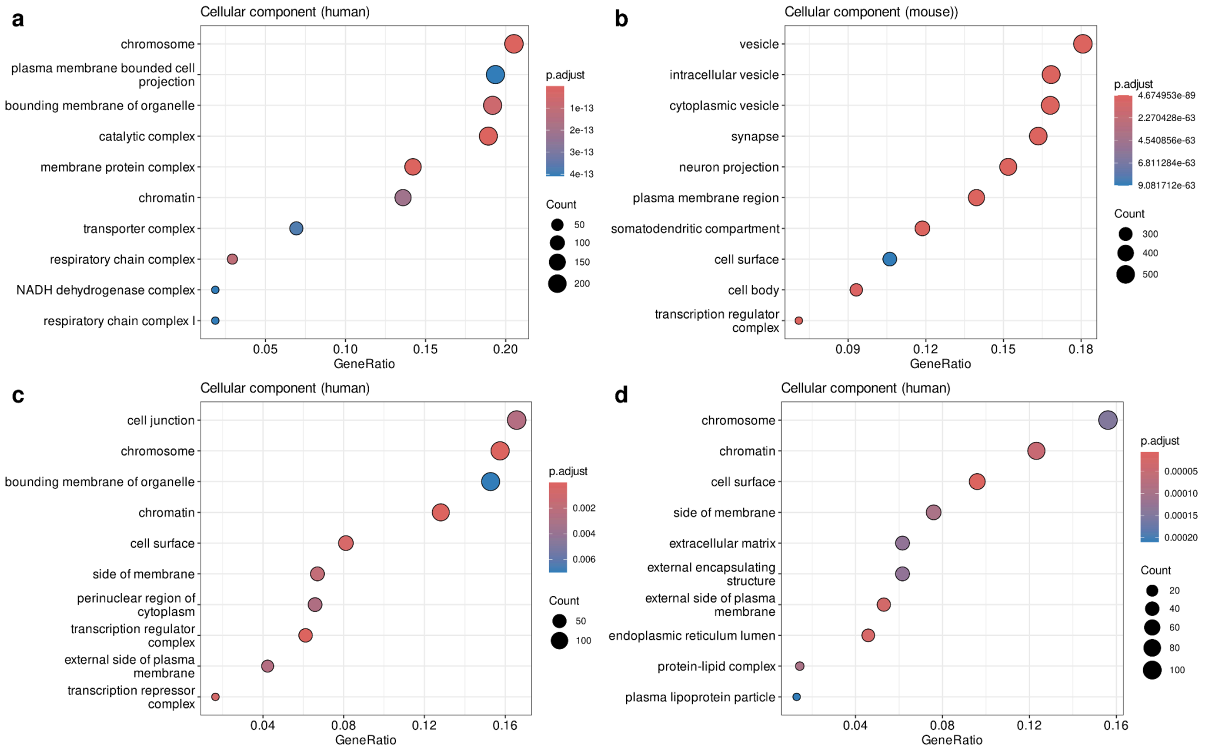

Extended Data Figure 10. Cellular components associated with high degree of pleiotropy. Shown are dot plots of the gene set enrichment analysis results of highly pleiotropic genes according to HPO (a), MGD (b), , pan-UK Biobank (c) and FinnGen (d) GWAS data. The analysis was performed against GO cellular component terms. Top 10 terms are shown in each case.

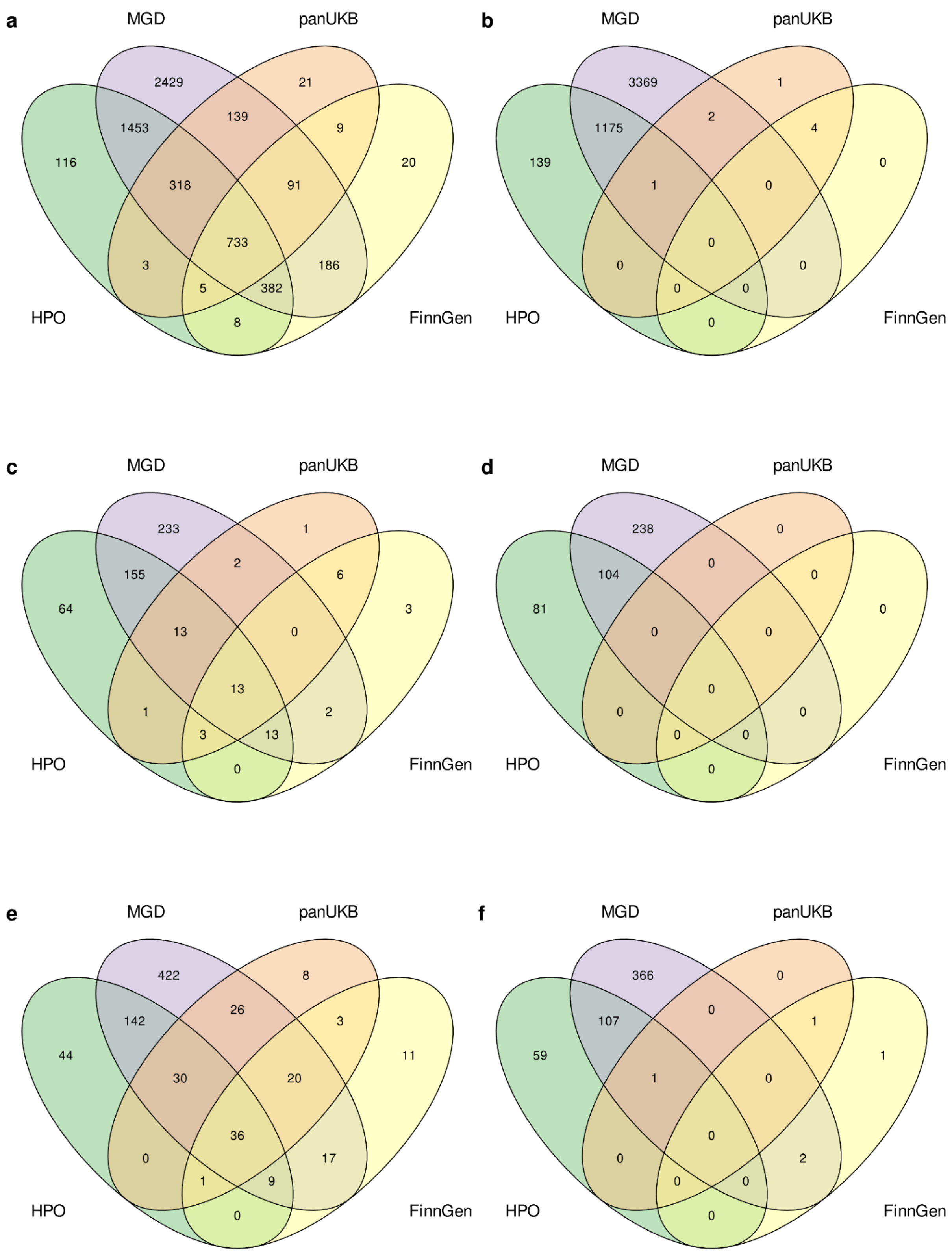

Extended Data Figure 11. Highly pleiotropic genes share a large fraction of enriched GO terms. Shown are Venn diagrams representing the overlap between sets of significantly enriched biological process (a-b), cellular component (c-d) and molecular function (e-f) terms for highly pleiotropic genes in the indicated datasets. On (b, d, f), genes having phenotype associations only in a given dataset (but not the other ones) were used for enrichment testing.

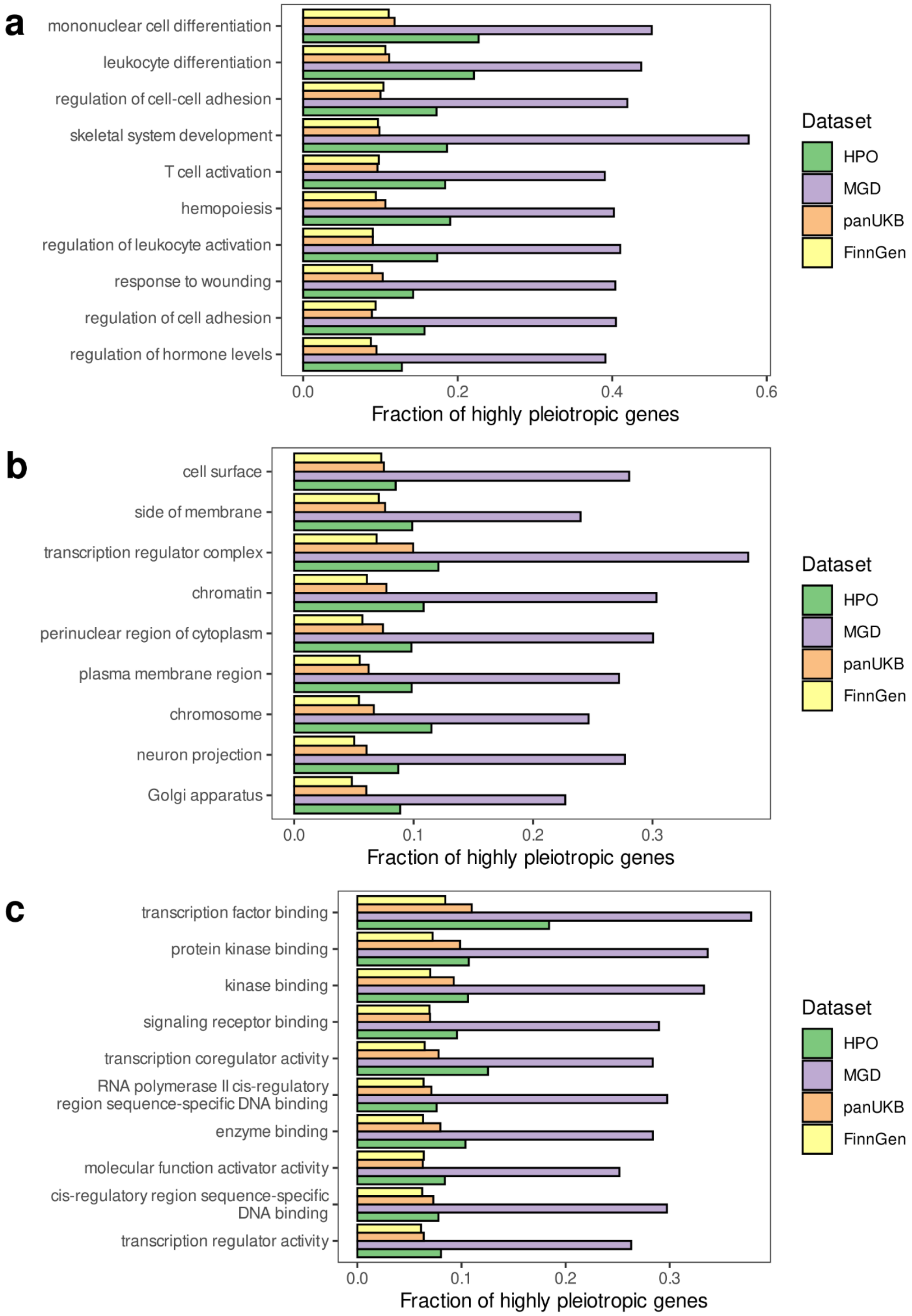

Extended Data Figure 12. Shared processes, cellular localization and molecular functions of pleiotropic genes. Shown are barplots of the fraction of highly pleiotropic genes among those annotated with a given biological process (a), cellular component (b), or molecular function (c) term from Gene Ontology. 10 terms with the highest proportions of pleiotropic genes are shown in each category.

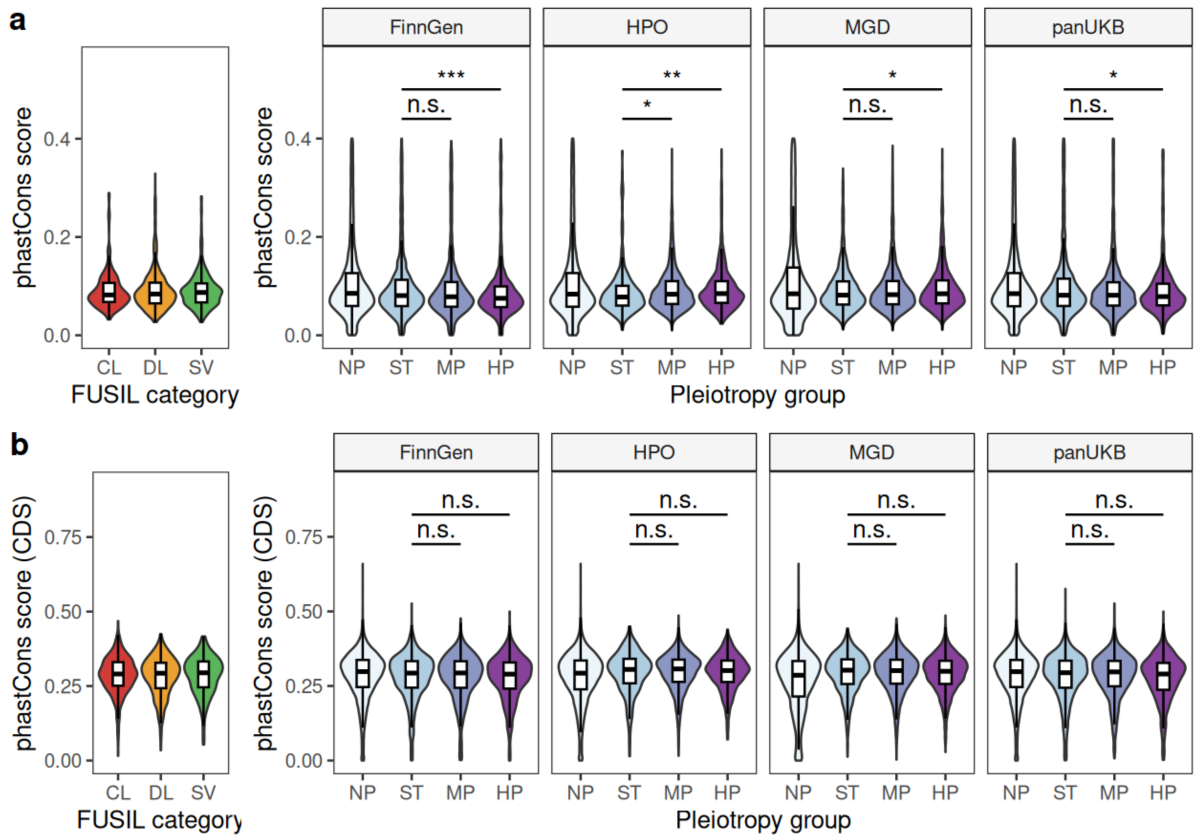

Extended Data Figure 13. Weak difference in the evolutionary conservation between genes in different pleiotropy groups. Shown are violin plots and box plots of the average (across the whole gene (a) or its coding sequence (b)) phastCons scores for genes in indicated groups and FUSIL essentiality categories. NP - genes with no associated phenotypes, ST - genes linked to a single trait or term, MP - moderately pleiotropic genes, HP - highly pleiotropic genes. FUSIL gene essentiality categories are abbreviated as CL (cellular lethal), DL (developmental lethal), and SV (subviable).

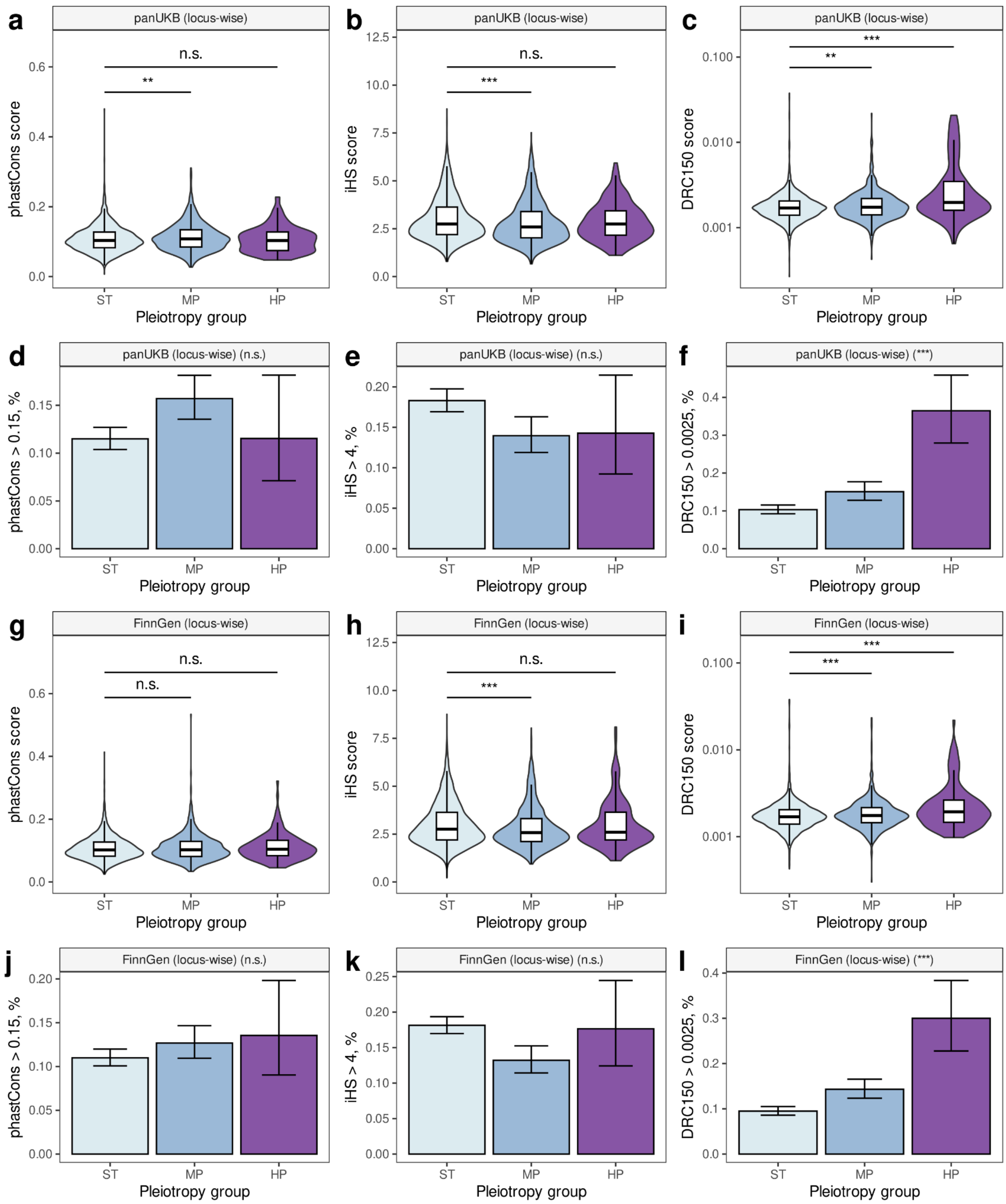

Extended Data Figure 14. Locus-wise metrics of positive and negative selection in the pan-UKB GWAS data. (a-c, g-i) Violin plots and box plots of average phastCons (a, g), maximum iHS (b, h) and DRC150 (c, i) values for each locus in the indicated pleiotropy groups in pan-UKB (a-c) and FinnGen (g-i) data. (d-f, j-l) Proportions of loci in the indicated groups that surpass a given cutoff for phastCons (d, j), iHS (e, k), or DRC150 (f, l) in pan-UKB and FinnGen data, respectively.

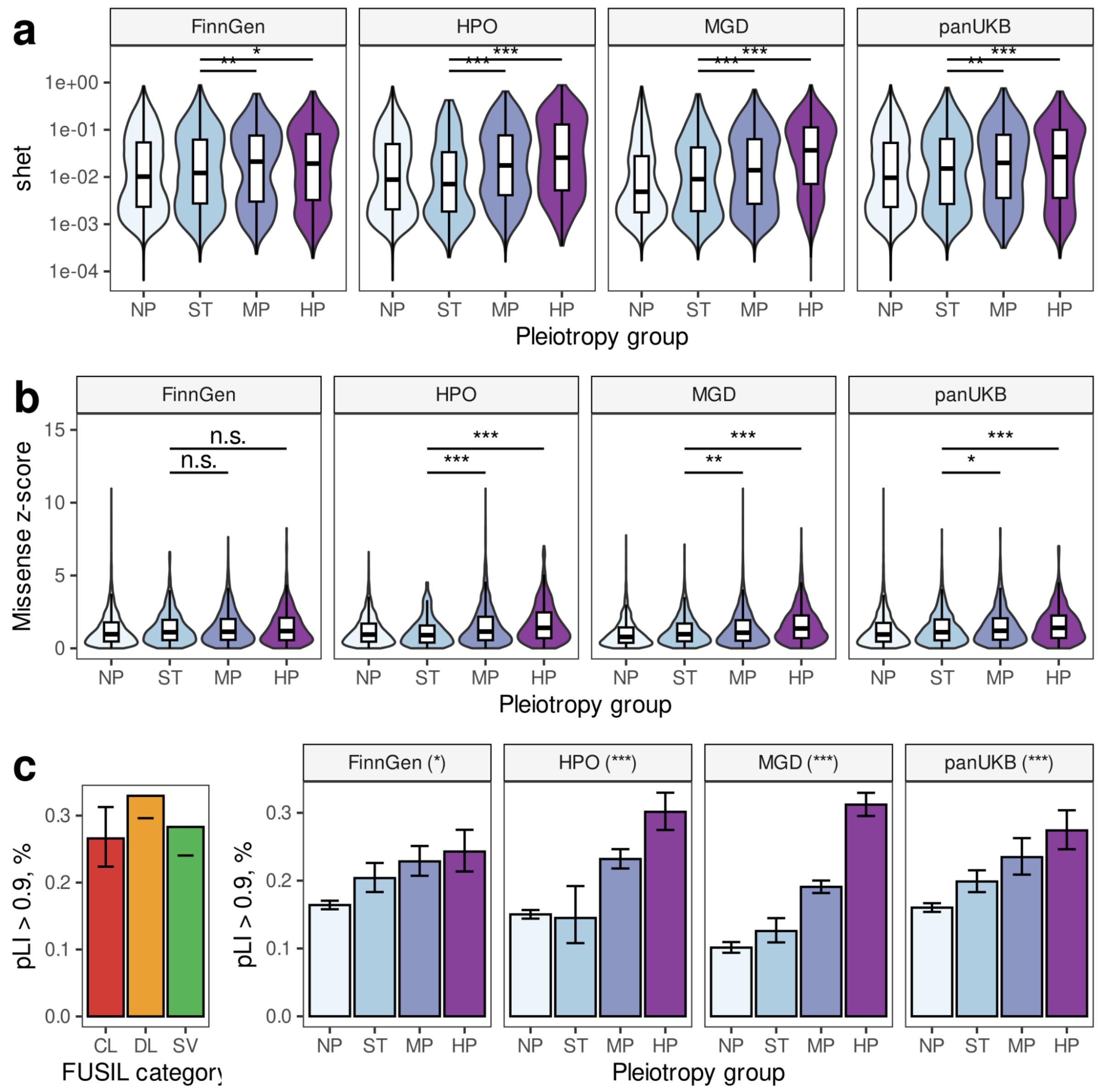

Extended Data Figure 15. Highly pleiotropic genes have a high degree of constraint against both pLoF and missense variation. Shown are violin plots and box plots of the selection coefficient against heterozygous protein-truncating variants (s_{het}) (a) and the Z-score of the observed-to-expected missense variant ratio (b) for indicated groups of genes. Values were taken from gnomAD v.2 data. On (c), bar plots represent the proportion of genes that are intolerant to loss-of-function variation according to the probability of loss-of-function intolerance (pLI > 0.9). NP - genes with no associated phenotypes, ST - genes linked to a single trait or term, MP - moderately pleiotropic genes, HP - highly pleiotropic genes. FUSIL gene essentiality categories are abbreviated as CL (cellular lethal), DL (developmental lethal), and SV (subviable).

.

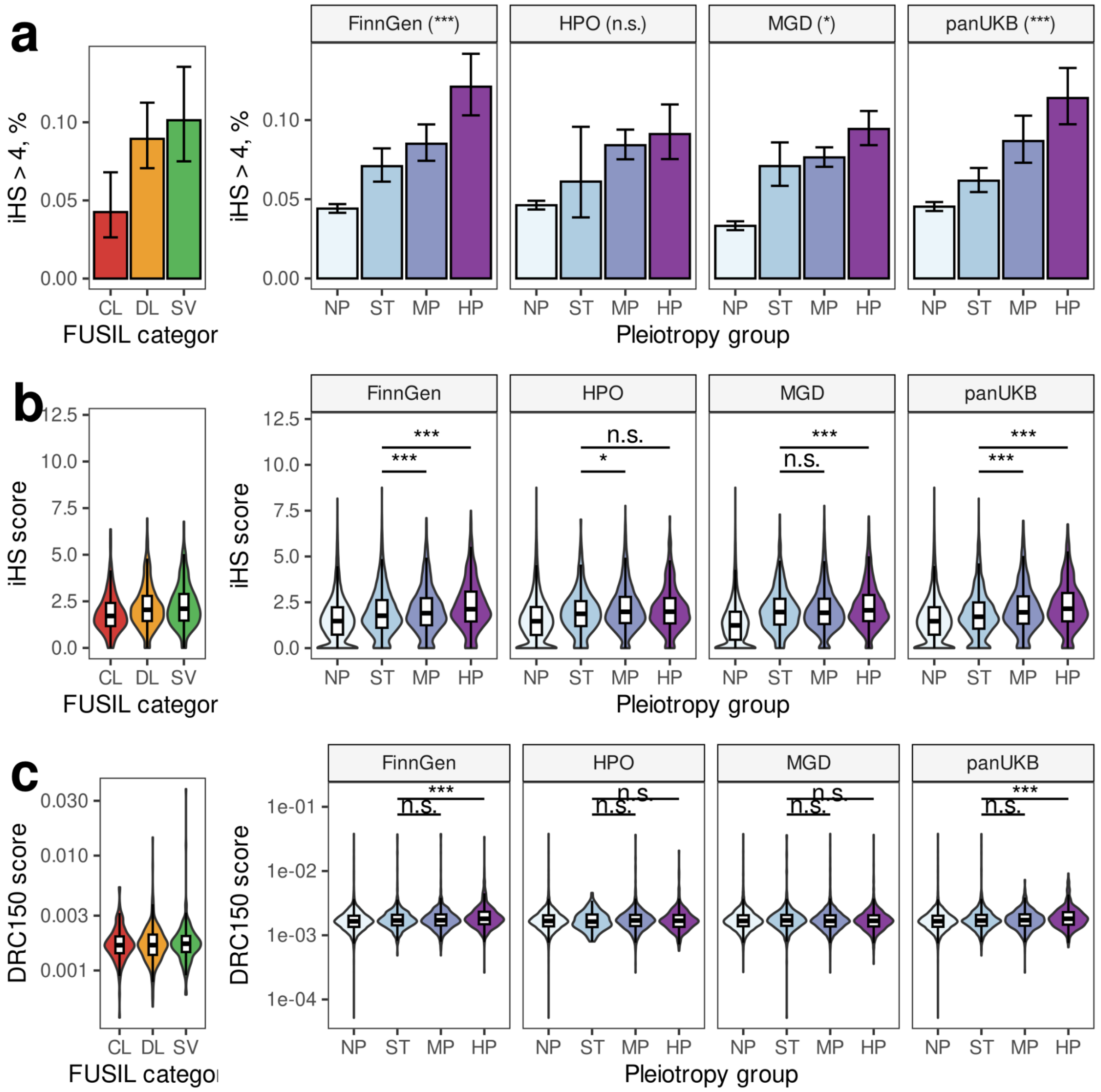

Extended Data Figure 16. Highly pleiotropic loci show an enrichment with recent positive selection signals. (a) Barplots representing the proportion of genes bearing at least SNP with iHS > 4 in indicated pleiotropy groups and gene essentiality categories. (b, c) Violin plots and box plots of the distribution of the gene-wise maximum iHS (b) and DRC150 (c) values for indicated gene groups. NP - genes with no associated phenotypes, ST - genes linked to a single trait or term, MP - moderately pleiotropic genes, HP - highly pleiotropic genes. FUSIL gene essentiality categories are abbreviated as CL (cellular lethal), DL (developmental lethal), and SV (subviable).

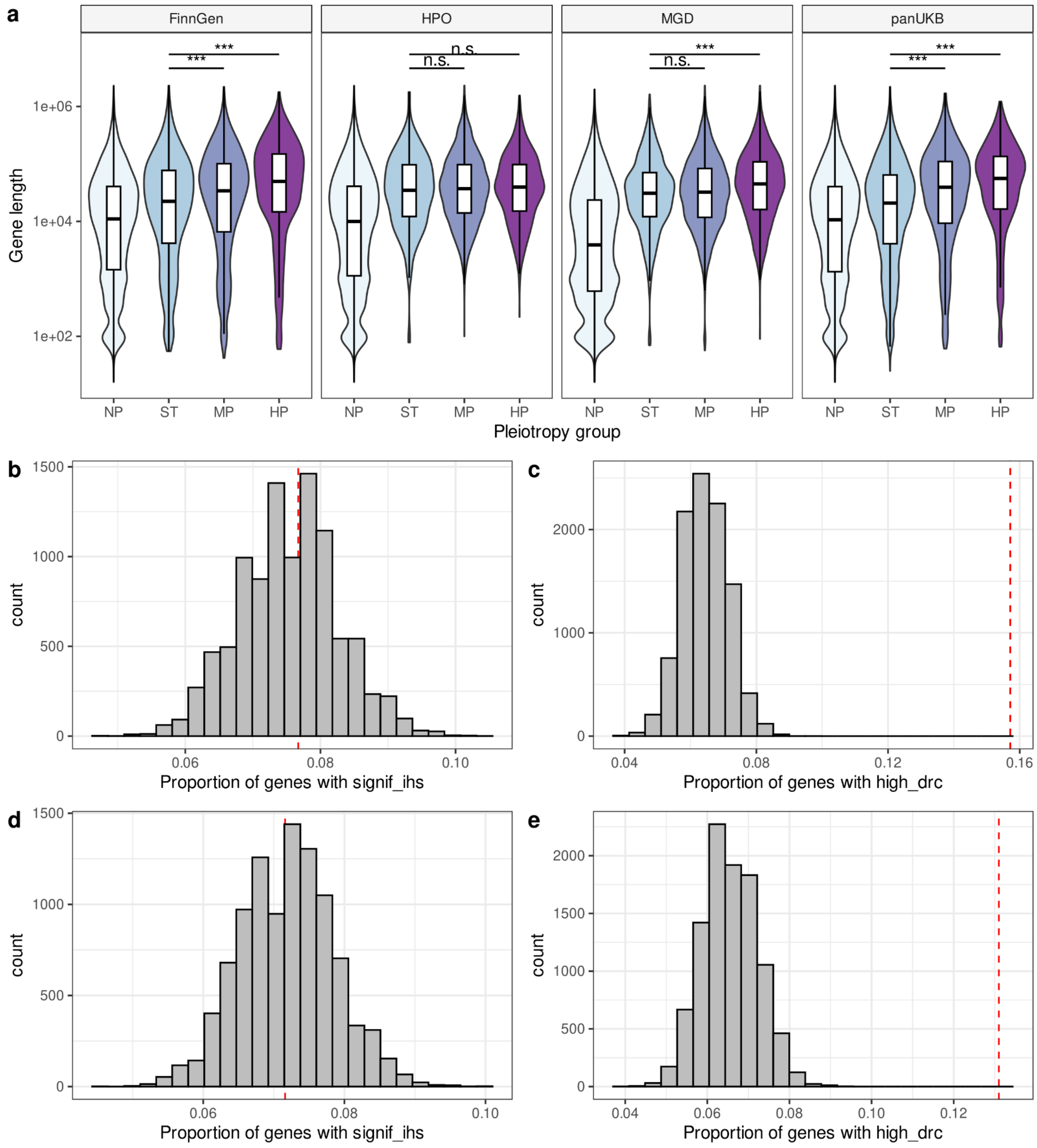

Extended Data Figure 17. Highly pleiotropic genes tend to be longer (especially in GWAS), but the length bias does not explain the observed enrichment of positive selection signals. (a) Violin plots and box plots showing the distribution of gene length (in log-scale) for the indicated pleiotropy groups. NP - genes with no associated phenotypes, ST - genes linked to a single trait or term, MP - moderately pleiotropic genes, HP - highly pleiotropic genes. (b-e) Histograms showing the distribution of the proportion of genes having an adjusted iHS p-value < 0.05 (b, d) or maximum DRC150 value > 0.0025 (c, e) in a random sample of genes with the length distribution matched to that of highly pleiotropic FinnGen (b-c) or pan-UK Biobank (d-e) genes. Dashed red line corresponds to the observed value in each case.

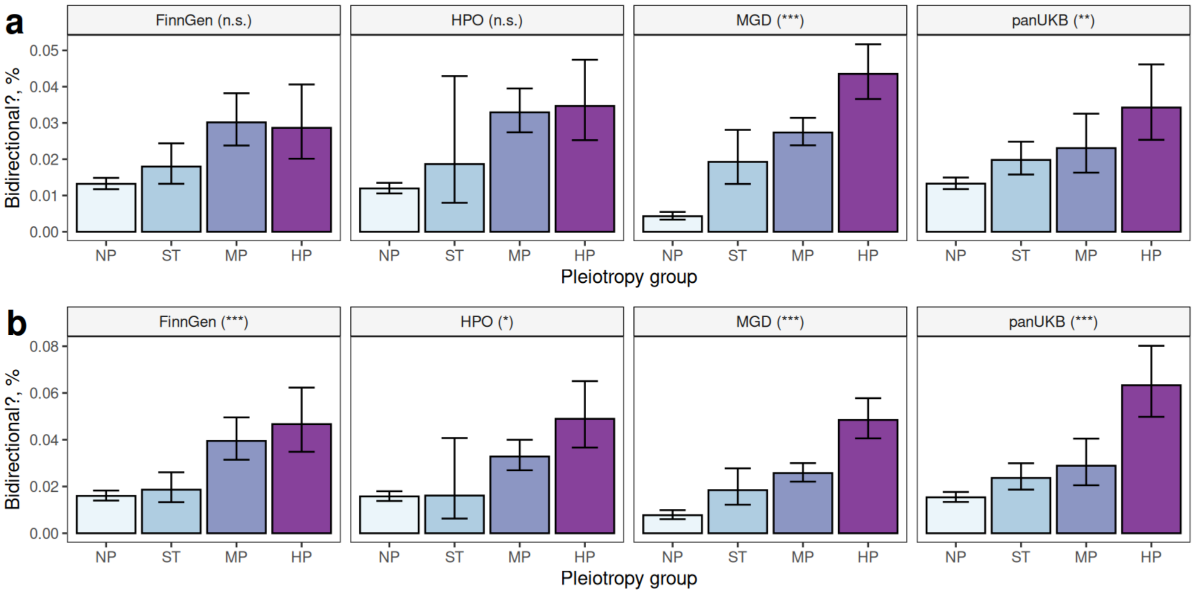

Extended Data Figure 18. Highly pleiotropic genes bear combined signals of positive and negative selection. Shown are bar plots representing the proportion of genes having a LOEUF value < 0.6 and either adjusted iHS p-value < 0.05 (a) or DRC150 > 0.0025 (b). NP - genes with no associated phenotypes, ST - genes linked to a single trait or term, MP - moderately pleiotropic genes, HP - highly pleiotropic genes.

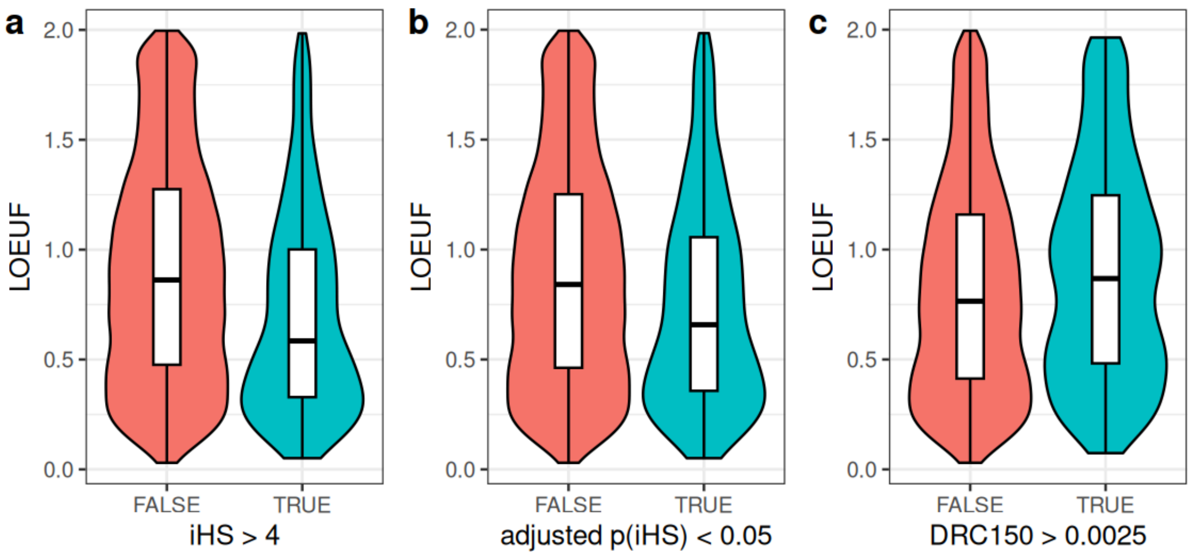

Extended Data Figure 19. Statistics used for recent positive selection detection (iHS and DRC150) demonstrate different dependence on gene constraint. Shown are violin plots and box plots of LOEUF values for genes with or without a recent positive selection according to three approaches (iHS > 4 (a), adjusted iHS p-value < 0.05 (b), DRC150 > 0.0025 (c)).

Supplementary Note

In this Supplementary Note, we will discuss several important questions that were not addressed in the main text of the manuscript, but are of importance for understanding the methodology of the study.

1. The choice of metric for assessing pleiotropy from phenotype ontology terms

Estimation of the *bona fide* (horizontal) pleiotropic effects is complicated by a variety of factors, one of which is correlations between traits. In the genome-wide association data, genetic correlation estimates enable grouping of traits with similar genetic basis to ensure that no highly correlated traits are counted as independent (see section 2 for a discussion of correlation-based clustering). For phenotype ontology term data gathered in monogenic trait setting, however, such estimates are not readily available.

Both Human Phenotype Ontology (HPO) and Mammalian Phenotype Ontology (MP) are hierarchically structured, with upper-level terms (e..g, "Abnormality of …") sometimes used as a proxy to measure pleiotropic effects (e.g., REFs). However, the distribution of gene-level term counts, while differing in the proportion of non-pleiotropic genes, have notable similarities in shape (Extended Data Figure 1). This poses the question regarding the validity of using upper-level PO terms as measures of horizontal pleiotropy. A similar question has been raised earlier in light of the Mouse Phenotype Consortium (MPC) data (REF Mammalian genome).

A possible way to remove redundancy in the data could be to use clustering of phenotype terms using data on associated genes. However, unlike complex trait clustering that can leverage genetic correlation estimates that are continuous in nature, clustering of phenotype ontology terms can be expected to suffer from a relatively low number of genes that are annotated with each specific term, thus making a distance metric used for clustering more discrete One might expect that, in such circumstance, unrelated terms could be spuriously grouped into a single cluster by virtue of having a single shared gene.

Nevertheless, we applied hierarchical clustering based on the Szymkiewicz–Simpson coefficient (overlap index) (a value of 1 - OI was used as a distance metric for clustering). The number of clusters was optimized using the silhouette score. Comparable number of clusters was identified for HPO and MP (5540 and 7138, respectively), with an average of 1.75 and 1.49 terms per cluster.

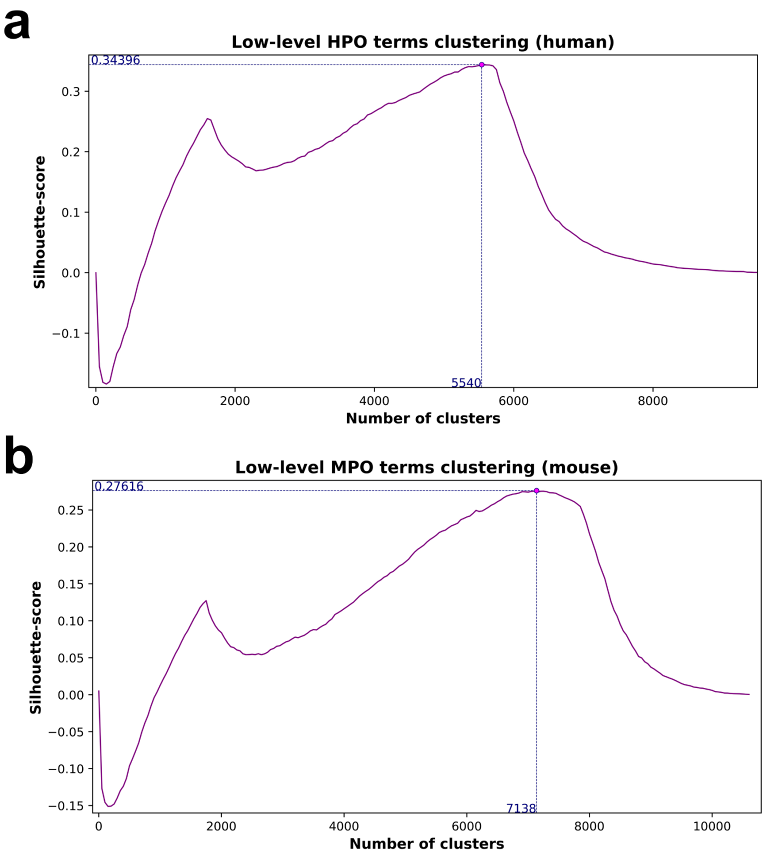

Figure SN1.1. Clustering of HPO and MP terms by similarity of the associated gene sets. Shown are line plots showing the silhouette score depending on the number of clusters for HPO (a) and MP (b) data.

We next used the obtained clustering of terms to re-calculate the gene-level degree of pleiotropy in both species. However, despite a shift in the distribution of the degree of pleiotropy when using clustered ontology terms compared to all ontology terms (Figure N1.2), the overall shape of the distribution was virtually unchanged in both human and mouse. The number of non-pleiotropic genes increased from 7 to 119 in humans, and from 909 to 1001 in mice. At the same time, this number was still smaller compared to the number of non-pleiotropic genes identified using upper-level MP terms as the measure of degree of pleiotropy (287 for humans and 1409 for mice). It is important to note that out of 119 genes with no pleiotropic effects according to the HPO term clusters, 76 (63.9%) remain non-pleiotropic according to upper-level MP term counts. For mice, this number amounts to 846 (84.5%).

Besides the number of non-pleiotropic genes, we also compared the median degree of pleiotropy per gene when using each of the approaches. Clustering of low-level ontology terms only slightly reduced the median pleiotropy compared to unclustered data in humans

(from 40 to 33), and did not introduce any change for the murine data (in both cases, 10 terms or term clusters per gene were observed). Expectedly, the median degree of pleiotropy was much lower with upper-level terms as a measure (11 - for humans, and 5 - for mice).

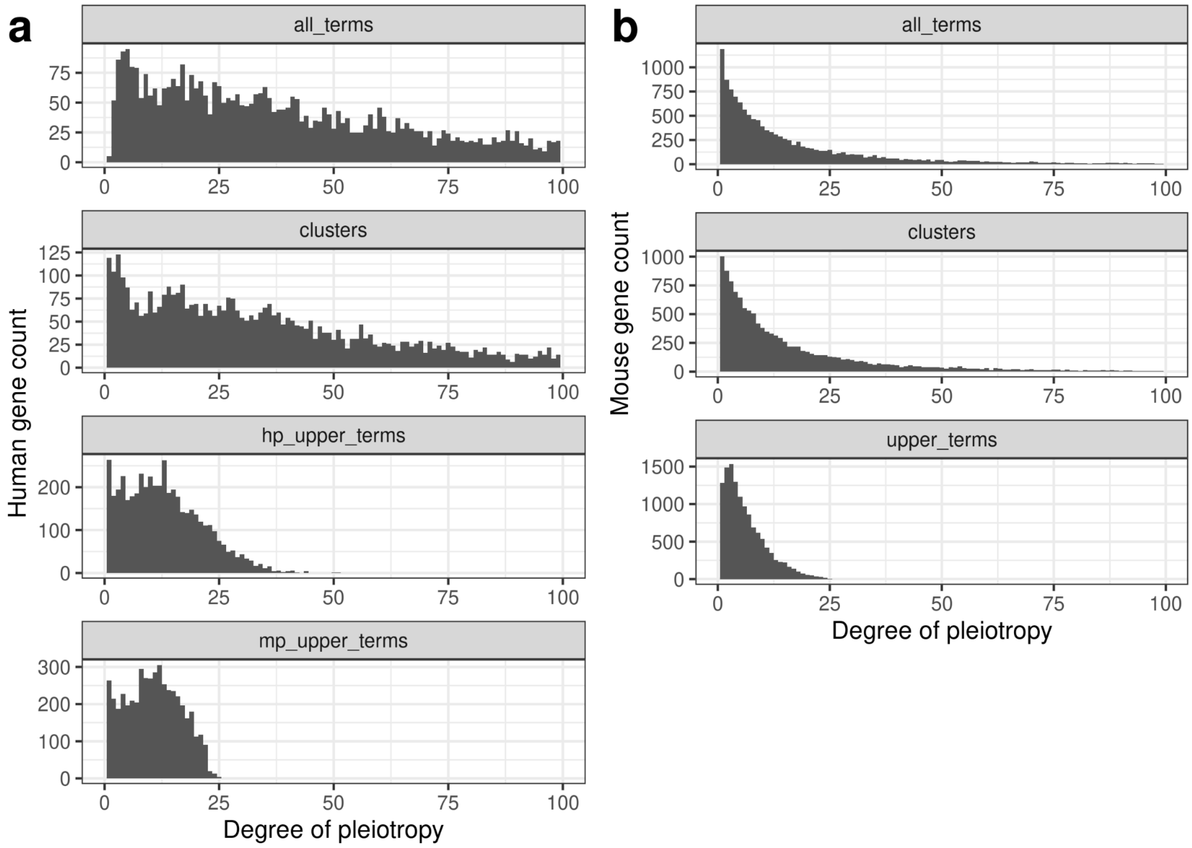

Figure SN1.2. The distributions of the per-gene degree of pleiotropy for human (left) and mouse (right) genes using either all ontology terms (top), or clusters of all ontology terms (middle), upper-level Mammalian Phenotype Ontology terms (bottom). For humans, an additional plot shows the number of upper-level HPO terms per gene before converting to MP vocabulary.

Besides the general similarity in the distribution shape, we have also evaluated the concordance between the number of upper-level HPO terms associated with a given gene and the number of matching upper-level MP terms after conversion. The results of the analysis showed that the values were expectedly highly correlated (Figure N1.3). More than 90% of genes (1013 out of 1109) that fell into the fourth quartile by their degree of pleiotropy using upper-level HPO terms were also in the fourth quartile of the distribution according to MP term count. The median degree of pleiotropy was also virtually the same (12 - with HPO terms, and 11 - with matching MP terms). Hence, we chose upper-level MP terms over upper-level HPO terms to enhance the comparison between the species.

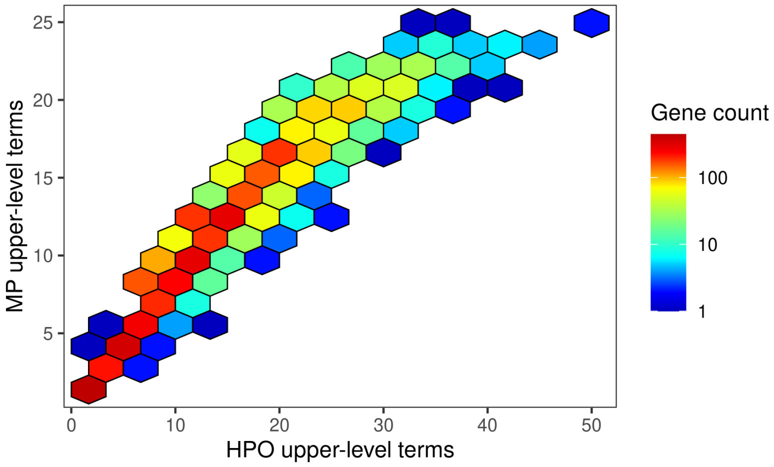

Figure SN1.3. Degree of pleiotropy estimated using upper-level HPO terms and mapped upper-level MP terms are strongly correlated. Shown is the hexagonal scatterplot of the number of terms associated with a given gene before and after converting HPO terms to MP terms. Color of the hexagon is proportional to the number of genes in the corresponding bin.

Taken together, these results suggest that clustering of ontology terms by similarity of the associated gene sets is less effective in reducing average degree of pleiotropy of a gene compared to grouping by upper-level terms. However, one has to bear in mind that this observation does not definitively prove that the upper-level terms are providing a better estimate of pleiotropy compared to clusters of lower-level terms.

2. Do closest genes well represent the genotype-to-phenotype associations observed at GWAS loci?

Another important issue pertinent to the analysis of gene-level pleiotropy from GWAS data is the identification of causal genes at the genome-wide significant loci. Three principal approaches can be utilized for solving this task: (i) picking the closest gene to each lead SNP identified after LD-based clumping of variants; (ii) using all genes that are located within a fixed size window from a lead SNP; and (iii) using statistical fine mapping or gene prioritization tools (reviewed in Uffelmann et al., 2021).

For pan-UK Biobank (pan-UKB) data, the latter approach requires access to the raw genotype information, making it hard to apply it to the summary data used in our work. Hence, we compared the other two options for pan-UKB data pre-processing. As expected, we found that much more genes appear as having at least one associated trait when using the second (locus-wide) approach compared to the closest gene approach (Figure SN2.1a). Thus,

a total of 16,569 genes were identified using the locus-wide approach compared to only 6469 for the closest gene approach. Similarly, a median of 2 associations per gene were present in the former dataset, corresponding to 46.8% of non-pleiotropic genes (in closest gene-based data, a median of 1 association per gene was observed, and 59.6% of genes were thus non-pleiotropic).

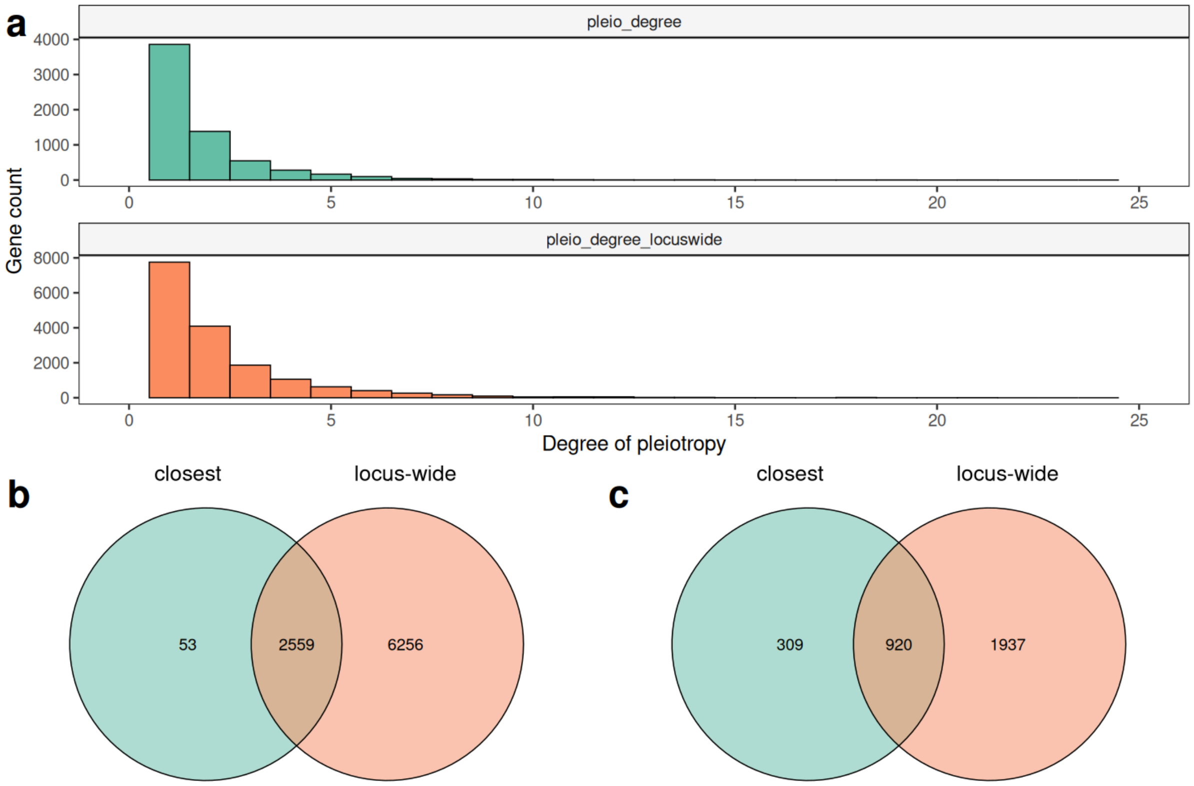

Figure SN2.1. (a) The distribution of the degree of gene-level pleiotropy calculated using closest gene-based and locus-wise (all genes within a 100,000 base pair window from lead SNP) GWAS data processing methods. (b, c). Venn diagrams showing the overlap between sets of pleiotropic (b) and highly pleiotropic (c) genes obtained using each method.

Expectedly, almost all of the genes that were identified as pleiotropic using the closest gene-based method were also pleiotropic in the locus-wise dataset (Figure SN2.1b). For highly pleiotropic genes, however, the overlap was lower, with as many as 309 genes designated as highly pleiotropic in the former dataset (Figure SN2.1c). This observation is likely driven by a lower cutoff for highly pleiotropic genes (3 trait clusters were required for closest gene-based data compared to 4 clusters for locus-wise data).

We next went on to evaluate whether the properties of pleiotropic genes identified using different data preprocessing strategies are similar, As shown in Figure SN2.2, the two strategies yielded similar results, with significant differences in expression pattern, GO term

counts, and gene-level constraint (measured using LOEUF) observed in both datasets (Figure SN2.2a,c,d). Of note, highly pleiotropic gene set obtained using locus-wise method demonstrated a significant increase in the number of protein-protein interactions (Figure SN2,2b); however, the differences in LOEUF were, to the contrary, more pronounced when using closest genes (Figure SN2.2d). Notably, the difference in the expression profile for was more pronounced when all genes within a 100,000 kbp window from an index variant were used for analysis. For example, median expression of the genes at highly pleiotropic loci was almost 7-fold higher compared to non-pleiotropic genes (only a 2.7-fold difference was observed for closest genes). At the same time, highly pleiotropic genes closest to lead GWAS SNPs were expressed at 5 TPM in as many as 10 tissues (median) compared to only 2 tissues for all genes at a locus.

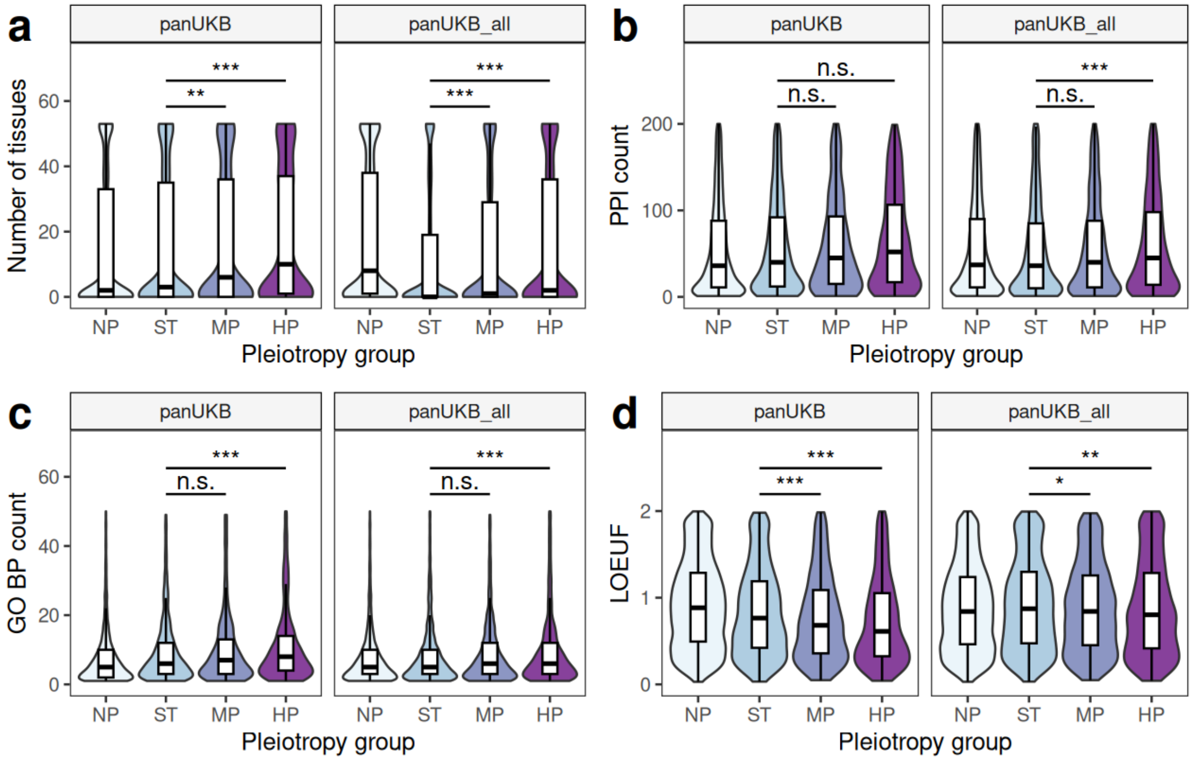

Figure SN2.2. Violin plots and box plots showing the distribution of gene properties for the given pleiotropy groups in GWAS data processed using closest gene picking (“panUKB”) or taking all genes with a 100,000 bp window (“panUKB_all”). Shown are the number of tissues with expression at the level of > 5 TPM according to GTEx v.8 (a), the number of protein-protein interactions (b), the number GO biological process (BP) terms (c), and the loss-of-function observed-to-expected upper fraction (LOEUF) according to gnomAD v.2. (d). *** - $p < 0.001$, ** - $p < 0.01$, n.s. - no significant differences in the Wilcoxon-Mann-Whitney test with Benjamini-Hochberg FDR adjustment.

Unlike pan-UKB, FinnGen provides the results of fine mapping of causal SNPs at the identified GWAS loci obtained using different pipelines. To evaluate if fine mapping of GWAS signal would significantly alter the conclusions made in our analysis, we set off to compare the results of pleiotropy analysis in FinnGen using the closest gene-based method or fine mapping results from the SuSIE pipeline (Zou et al., 2023).

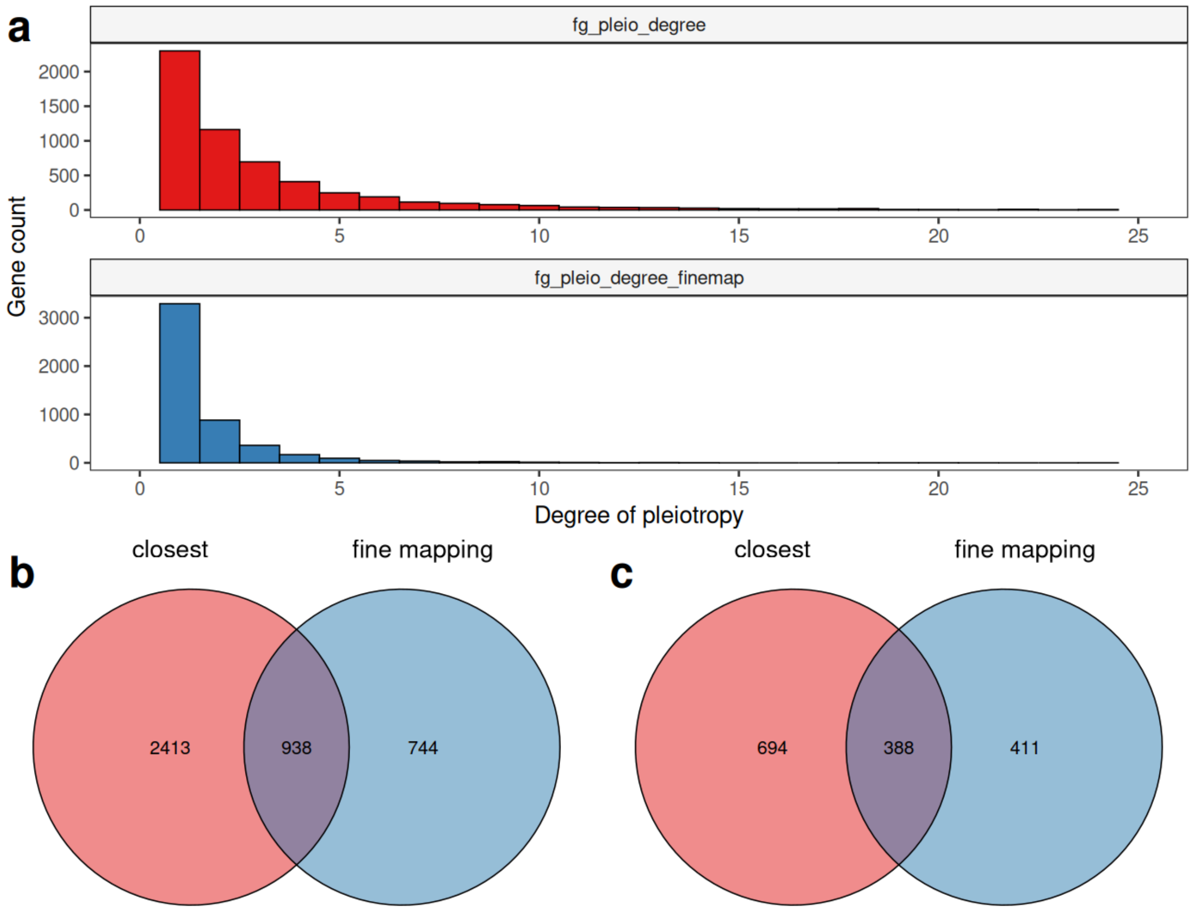

Figure SN2.3. (a) The distribution of the degree of gene-level pleiotropy calculated using closest gene-based and fine-mapping-based FinnGen GWAS data. (b, c). Venn diagrams showing the overlap between sets of pleiotropic (b) and highly pleiotropic (c) genes obtained using each method.

Of note, significantly fewer genes bore trait associations if using fine mapping results for the analysis (4971 vs. 5650, Figure SN2.3a). This difference likely stems from a substantial proportion of GWAS loci for which no intragenic variant was identified during fine mapping. In contrast to the different methods employed for pan-UKB data processing, closest gene-based and fine mapping-based approaches showed markedly lower overlap of sets of pleiotropic genes (Figure SN2.3b-c). Thus, only 55.7% of pleiotropic genes identified using fine mapping were also pleiotropic based on closest gene analysis. For highly

pleiotropic genes, the proportion was slightly lower (48.6%). These results emphasize that closest gene-based analysis, while providing a more comprehensive coverage of genes, is likely to miss the actual causal gene in a significant proportion of cases.

A substantial difference in the sets of pleiotropic genes identified using the two methods led to some discrepancies in the results of functional gene property analysis. For example, highly pleiotropic genes had significantly broader expression compared to non-pleiotropic ones only when using closest gene picking (Figure SN2.4a). At the same time, significant differences in the number of PPIs per gene were identified when using fine mapping data, but not the closest gene-based method (Figure SN2.4b). We anticipate that these differences result from a higher proportion of well-established protein-coding genes in fine mapping results, which is consistent with a much broader expression of genes identified using fine mapping, even at non-pleiotropic loci (Figure SN2.4a). For other metrics tested, both closest gene-based and fine mapping-based methods yielded similar results (Figure SN2.4c-d).

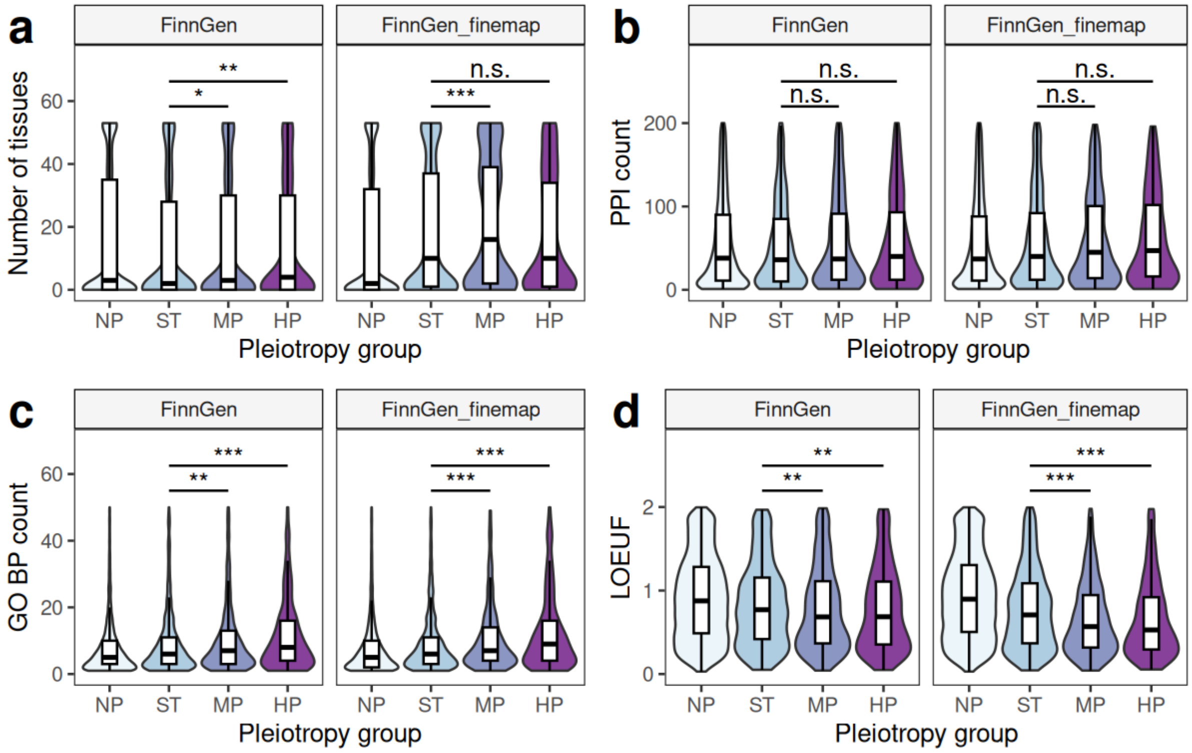

Figure SN2.4. Violin plots and box plots showing the distribution of gene properties for the given pleiotropy groups in FinnGen GWAS data processed using closest gene picking or fine mapping. Shown are the number of tissues with expression at the level of > 5 TPM according to GTEx v.8 (a), the number of protein-protein interactions (b), the number GO biological process (BP) terms (c), and the loss-of-function observed-to-expected upper fraction (LOEUF) according to gnomAD v.2. (d). *** - $p < 0.001$, ** - $p < 0.01$, n.s. - no significant

differences in the Wilcoxon-Mann-Whitney test with Benjamini-Hochberg FDR adjustment. NP - genes with no associated phenotypes, ST - genes linked to a single trait or term, MP - moderately pleiotropic genes, HP - highly pleiotropic genes.

Besides, different approaches to FinnGen data processing yielded slightly different results in positive selection analysis. Thus, the proportion of genes bearing a significant positive selection signal according to the iHS value was higher for highly pleiotropic genes irrespective of the analysis method employed (Figure SN2.5a), and the proportion of such genes was markedly higher when using fine mapping. At the same time, when the DRC150 value was employed instead of iHS, significant differences between highly pleiotropic and non-pleiotropic genes were only identified in closest gene-based data (Figure SN2.5b).

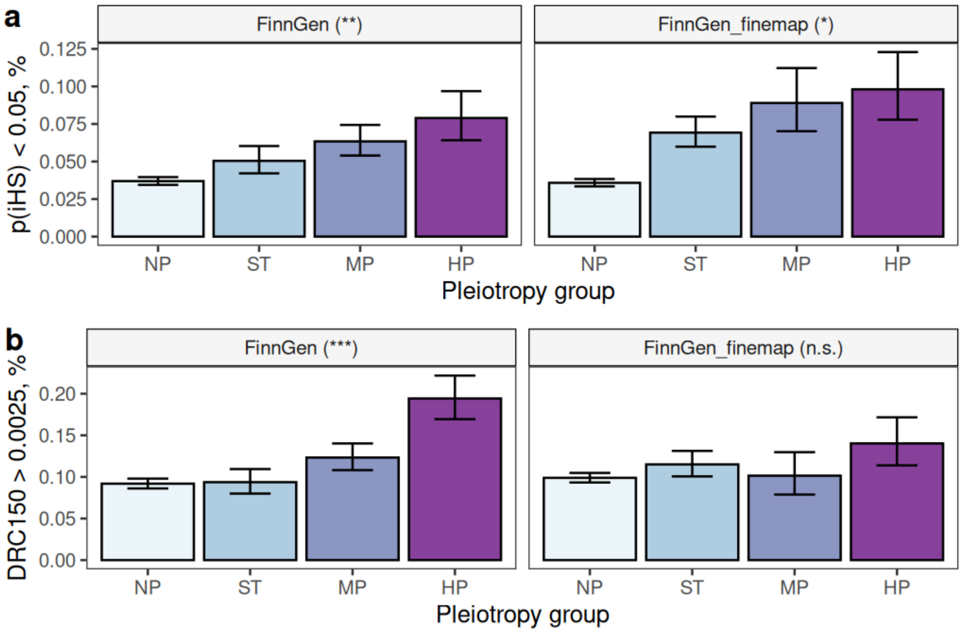

Figure SN2.5. Bar plots showing the proportion of genes bearing positive selection signals in given pleiotropy groups for FinnGen GWAS data processed using closest gene-based or fine mapping-based methods. Shown are results for iHS (based on p-value of gene-wise maximum score, see main text for details) (a) and DRC150 (b). Error bars correspond to 95% confidence intervals based on binomial distribution. NP - genes with no associated phenotypes, ST - genes linked to a single trait or term, MP - moderately pleiotropic genes, HP - highly pleiotropic genes.

Taken together, the results presented above show that both locus-wise and closest gene-wisse analysis methods provide similar insights into the mechanistic basis of pleiotropic effects, and small differences observed when comparing the two datasets side-by-side can be attributed to the different cutoffs used for highly pleiotropic gene identification. The locus-wise approach, however, can be expected to yield a significant number of genes with false positive associations and spuriously pleiotropic genes that are located in the vicinity of

several distinct causal genes. Hence, the closest gene-based approach was used in the main part of the analysis presented in the article.

3. Pleiotropy analysis via clustering of complex traits compared to maximum independent set of traits

Previously, we have shown that clustering of traits by their phenotypic correlation is important to reduce spurious pleiotropy resulting from a large number of highly correlated traits in cohort datasets such as UK Biobank (Shukov et al., 2020). In particular, clustering allows the reduction of the proportion of the genome covered by pleiotropic loci from more than 60% (as reported by Watanabe et al., 2019) to ~5% (178.4 Mbp of the sequence).

In the pan-UK Biobank study (Karczewski et al., 2024), the authors have constructed the so-called maximum independent set of traits with low levels of correlation ($r^2 < 0.1$). In the data release used in our analysis, this set of traits comprised 150 traits of different types, 86 of which were continuous biomarkers traits used for further comparison. While such an independent set of traits should be perfectly suited to reduce the vertical pleiotropy in the data, one might expect that picking a single trait from a highly correlated cluster may significantly reduce the number of loci with genome-wide associations in the dataset. Hence, we compared the results of pleiotropy analysis using the maximum independent set of traits or the trait clustering used in the main part of our analysis.

First, we compared the distribution of the gene-level degree of pleiotropy in the two datasets. Expectedly, only 6972 gene-trait associations were detected using the maximum independent set, compared to 12,065 using the clustered dataset. The average degree of pleiotropy was slightly lower for the maximum independent trait set (1.73 compared to 1.87), and the proportion of pleiotropic genes was also slightly decreased (1368/4034, 33.9% compared to 2612/6469, 40.4%). The distribution of the degree of pleiotropy in the two data sources is shown on Figure SN3.1a. Besides, the degree of pleiotropy was highly correlated between the two datasets (Spearman's $\rho = 0.63$ $p < 0.001$). 146 genes were uniquely pleiotropic in the maximum independent trait set (Figure SN3.1b), and only 118 genes were uniquely highly pleiotropic in the maximum independent set, with 526 highly pleiotropic genes overlapping between the two datasets (Figure SN3.1c).

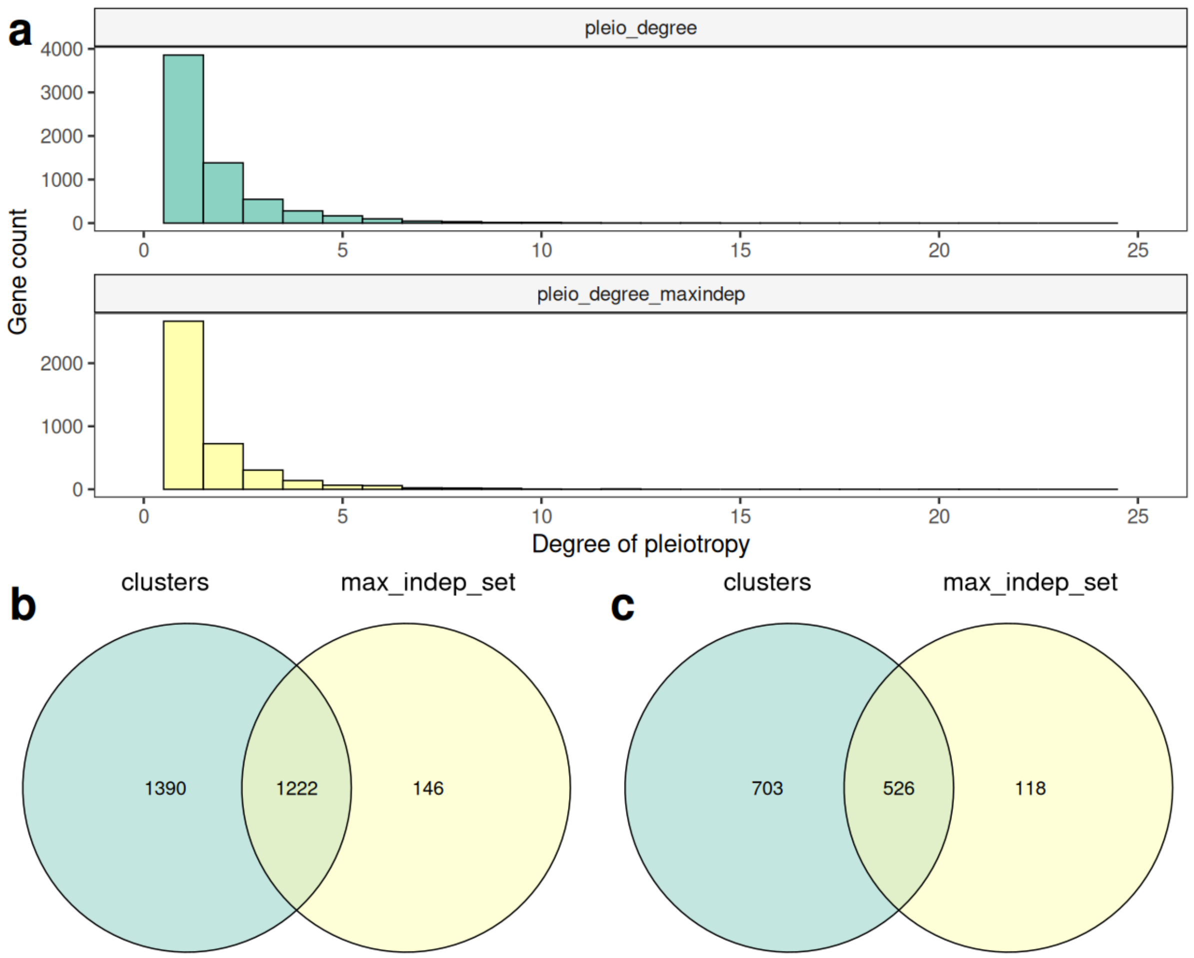

Figure SN3.1. (a) The distribution of the degree of gene-level pleiotropy calculated using clusters of correlated complex traits (top) or the maximum independent set of complex traits (bottom). (b, c). Venn diagrams showing the overlap between sets of pleiotropic (b) and highly pleiotropic (c) genes obtained using each method.

Given the subtle differences in the proportion of pleiotropic genes and the average degree of pleiotropy, we next moved on to test if the same differences in gene properties will be observed when using the maximum independent set of traits to measure pleiotropy in the complex trait domain. The analysis showed that, for all features that were evaluated, grouping of genes based on pleiotropy in the maximum independent trait set yielded the same results as the clustering-based approach (Figure SN3.2). Thus, significant differences remained between highly pleiotropic and non-pleiotropic genes in terms of the breadth of expression profile (number of tissues with gene expression at at least 5 TPM (Figure SN3.2a)), number of biological process terms (Figure SN3.2c), and evolutionary constraint level (Figure SN3.2d). For the number of protein-protein interactions, no significant differences were observed in either case (Figure SN3.2b). The only difference in results between the two

analysis approaches was a universally lower level of statistical significance when comparing various features between groups of genes (Figure SN3.2).

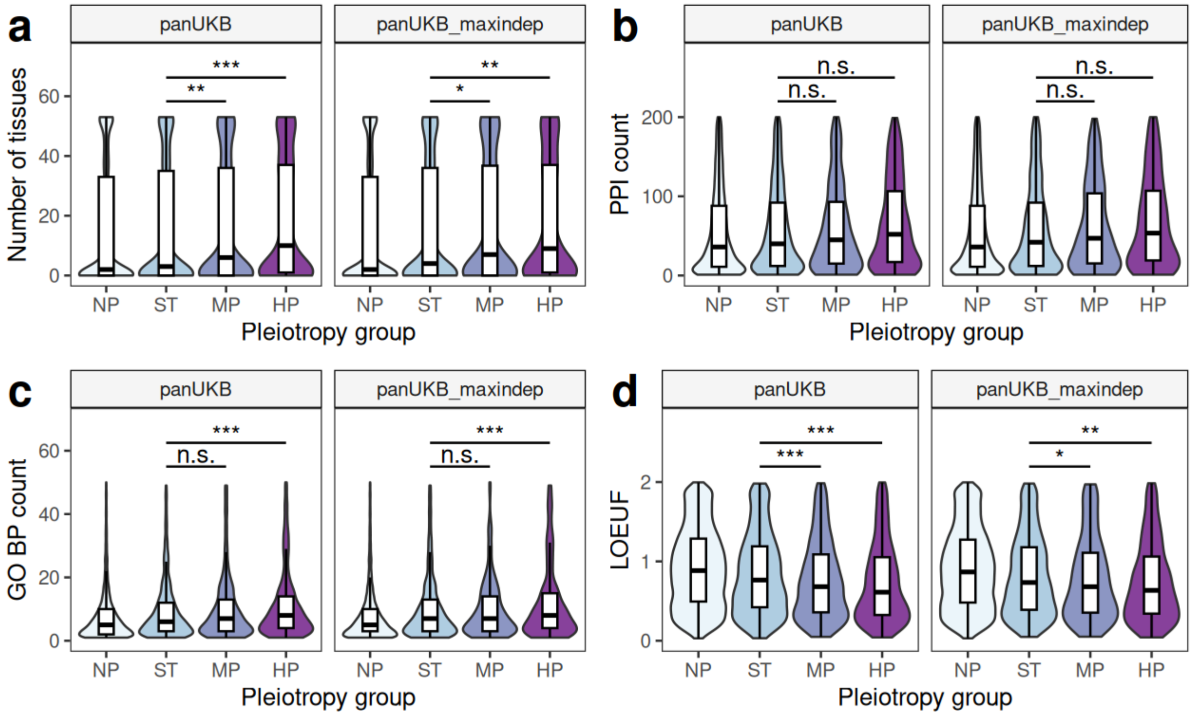

Figure SN3.2. Violin plots and box plots showing the distribution of gene properties for the given pleiotropy groups in indicated datasets. Shown are the number of tissues with expression at the level of > 5 TPM according to GTEx v.8 (a), the number of protein-protein interactions (b), the number GO biological process (BP) terms (c), and the loss-of-function observed-to-expected upper fraction (LOEUF) according to gnomAD v.2. (d). *** - $p < 0.001$, ** - $p < 0.01$, n.s. - no significant differences in the Wilcoxon-Mann-Whitney test with Benjamini-Hochberg FDR adjustment.

Taken together, the results presented in this section show that the maximum independent set of traits provided by the authors of the pan-UKB study does not provide any noticeable advantage over the approach based on trait clustering, and can even reduce the significance of certain trends. Hence, the method based on trait clusters was used as a standard one throughout the main part of the manuscript.

4. Patterns of pleiotropy in murine quantitative trait loci (QTL) data

In the main part of the analysis, we have drawn conclusions regarding the pleiotropic effects of genes on complex traits using two large compendia of genome-wide association studies' results, pan-UKB and FinnGen. While these two resources provide remarkable

coverage across the human phenome, the extent to which the results obtained can be generalized to other species remains unclear. Explicit evaluation of the patterns of complex trait pleiotropy in non-human mammalian species is complicated due to the lack of genome-wide association data for other species obtained from large cohorts.

To circumvent this limitation, we attempted to employ data about murine quantitative trait loci (QTL) available from the Mouse Genome Database (https://www.informatics.jax.org/downloads/reports/MGI_QTLAllele.rpt). While this dataset is still less rich compared to either of the human complex trait datasets used in the analysis, this resource contains a large amount of statistical associations between various traits and loci. From the total of 13,729 entries in the QTL dataset, clearly defined genomic coordinates were available only for 6179; of these, only 1938 (31.3%) had phenotype information represented in the form of MP ontology terms. An average of 1.48 upper-level MP terms were associated with these QTLs (with a median of 1 association per locus). Expectedly, the form of the distribution of the degree of pleiotropy was similar to the one observed for human complex traits (Figure SN4.1a).

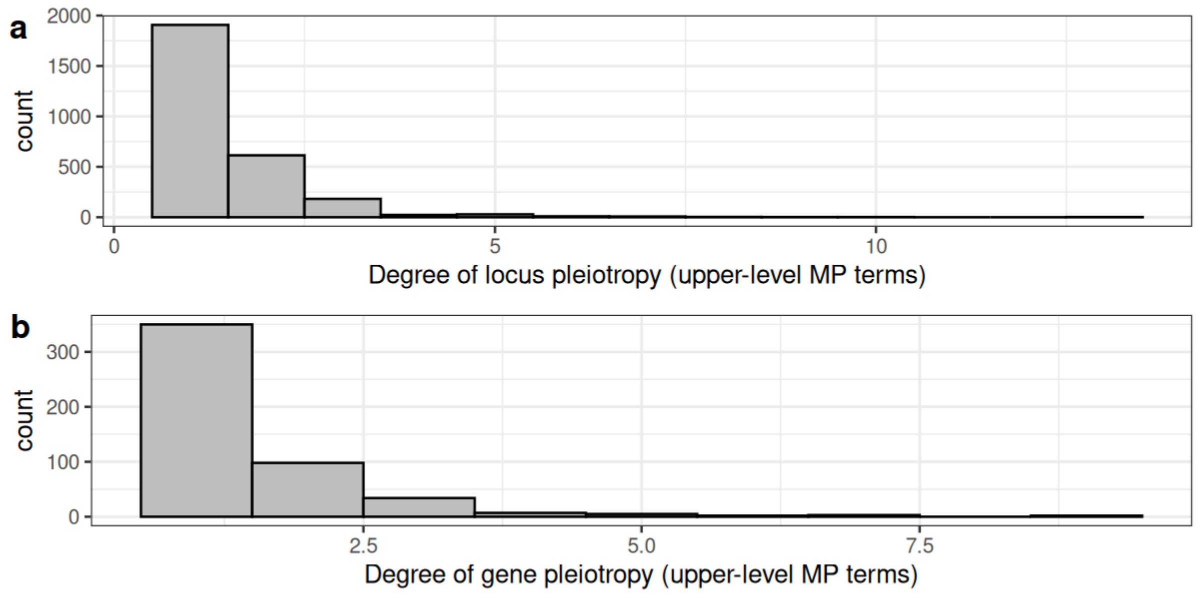

Figure SN4.1. Histogram showing the distribution of the degree of pleiotropy (estimated using upper-level MP terms) for 1938 loci with defined genomic coordinates (a) and for XXX murine genes that were unambiguously mapped to these loci (b).

Having calculated the degree of pleiotropy for QTLs with known mapping to the mouse genome, we next set off to identify genes at these loci. Unfortunately, mapping of loci to genes was obstructed by the heavy skew of the QTL length distribution (for example,

QTLs in the dataset had a median length of 194 bp, and an average length of 14,595,242 bp; with as many as 797 loci spanning more than 100,000 bp). Such a form of the distribution led to a large fraction of QTLs that overlapped no protein coding genes as well as loci that spanned more than 10 genes (894 of the 1960 loci spanning at least 1 gene, 45.6%). Only 501 of loci that had both phenotype annotations and known genomic coordinates could be mapped to a single gene. When the degree of pleiotropy distribution was recalculated using these genes, most genes still harbored only 1 associated MP term (Figure SN4.1b).

Having obtained QTL-based degree of pleiotropy estimates for the 501 murine genes, we decided to compare these estimates with the ones from the Mendelian trait dataset. Out of 145 genes that were pleiotropic in the murine QTL data, 107 (73.8%) were also pleiotropic according to the main MGD dataset; however, no statistically significant correlation was observed between the degree of pleiotropy estimated using each type of data (Spearman's ρ = -0.03, p-value > 0.05; Figure SN4.2).

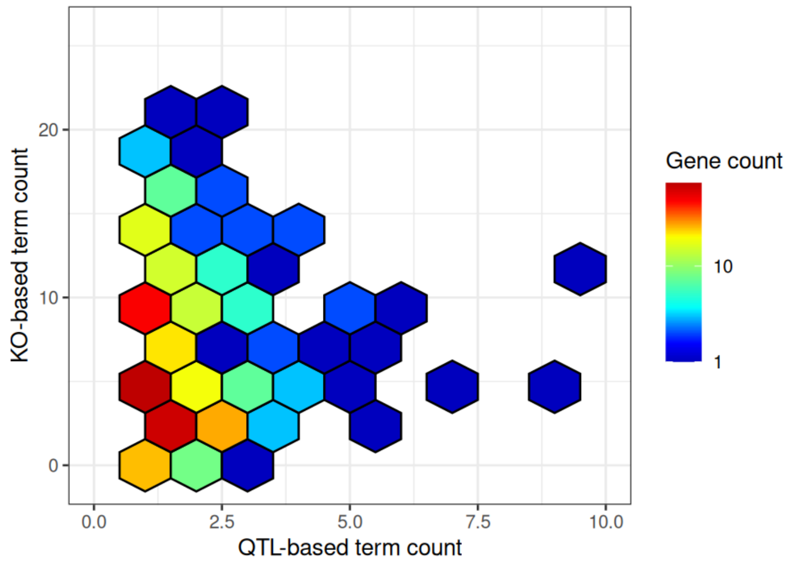

Figure SN4.2. A hexagonal plot showing the correspondence between the degree of pleiotropy (estimated using the number of associated upper-level MP terms) estimated using standard marker data and QTL data.

When the QTL data were used for functional gene property analysis, no significant differences in gene properties were observed depending on the degree of pleiotropy. (data not shown) While these results do not support the findings made using human complex trait data, it appears likely such a discrepancy stems from the very low number of pleiotropic (and, especially, highly pleiotropic) genes in the dataset.

Supplementary references

1. Uffelmann, E., Huang, Q.Q., Munung, N.S. *et al.* Genome-wide association studies. *Nat Rev Methods Primers* **1**, 59 (2021).
